# Eomesodermin-expressing CD4 Th cells and association with multiple sclerosis progression and brain atrophy

**DOI:** 10.1093/braincomms/fcaf415

**Published:** 2025-10-27

**Authors:** Marielena Bongert, Emelie Schönauer, Paulina Trendelenburg, Ulas Ceylan, Theodoros Ladopoulos, Katja Vohl, David Bratek, Annika Mattukat, Léon Beyer, Klaus Gerwert, Takashi Yamamura, Carsten Lukas, Ingo Schmitz, Ruth Schneider, Ilya Ayzenberg, Ralf Gold, Simon Faissner

**Affiliations:** Department of Neurology, Ruhr-University Bochum, St. Josef-Hospital, Bochum 44791, Germany; Department of Neurology, Ruhr-University Bochum, St. Josef-Hospital, Bochum 44791, Germany; Department of Neurology, Ruhr-University Bochum, St. Josef-Hospital, Bochum 44791, Germany; Department of Neurology, Ruhr-University Bochum, St. Josef-Hospital, Bochum 44791, Germany; Department of Neurology, Ruhr-University Bochum, St. Josef-Hospital, Bochum 44791, Germany; Institute of Neuroradiology, Ruhr-University Bochum, St. Josef-Hospital, Bochum 44791, Germany; Department of Neurology, Ruhr-University Bochum, St. Josef-Hospital, Bochum 44791, Germany; Department of Neurology, Ruhr-University Bochum, St. Josef-Hospital, Bochum 44791, Germany; Institute of Neuroradiology, Ruhr-University Bochum, St. Josef-Hospital, Bochum 44791, Germany; Department of Neurology, Ruhr-University Bochum, St. Josef-Hospital, Bochum 44791, Germany; Center for Protein Diagnostics (ProDi), Biospectroscopy, Ruhr-University Bochum, Bochum 44801, Germany; Center for Protein Diagnostics (ProDi), Biospectroscopy, Ruhr-University Bochum, Bochum 44801, Germany; Department of Immunology, National Institute of Neuroscience, NCNP, Tokyo 187-8502, Japan; Institute of Neuroradiology, Ruhr-University Bochum, St. Josef-Hospital, Bochum 44791, Germany; Department of Molecular Immunology, Ruhr-University Bochum, Bochum 44801, Germany; Department of Neurology, Ruhr-University Bochum, St. Josef-Hospital, Bochum 44791, Germany; Institute of Neuroradiology, Ruhr-University Bochum, St. Josef-Hospital, Bochum 44791, Germany; Department of Neurology, Ruhr-University Bochum, St. Josef-Hospital, Bochum 44791, Germany; Department of Neurology, Ruhr-University Bochum, St. Josef-Hospital, Bochum 44791, Germany; Department of Neurology, Ruhr-University Bochum, St. Josef-Hospital, Bochum 44791, Germany

**Keywords:** multiple sclerosis, progression, neuroprotection, T cells, biomarker

## Abstract

Multiple sclerosis is a chronic inflammatory disease affecting the central nervous system. While treatment made huge advances, progression remains a challenge. Eomesodermin^+^ Th cells are associated with cytotoxicity, neuroinflammation and disease progression in secondary progressive multiple sclerosis. We performed a prospective longitudinal study over one year to investigate the role of Eomesodermin^+^ T cells in multiple sclerosis progression and neurodegeneration. Patients underwent detailed clinical assessment and immunophenotyping. In a subcohort of patients, cross-sectional magnetic resonance imaging using voxel-based morphometry was performed. Frequencies of Eomesodermin^+^ Th cells could differentiate between patients with secondary and primary progressive multiple sclerosis and correlated with B-cells in secondary progressive multiple sclerosis. Secondary progressive patients experiencing a subjective worsening of disease activity showed higher baseline frequencies of Eomesodermin^+^ Th cells. Higher baseline frequencies of Eomesodermin^+^ Th cells predicted prospective one-year disability progression in a small group of secondary progressive patients. Conclusively, voxel-based morphometry analysis revealed pronounced infratentorial brain atrophy in a sub-cohort of patients with higher Eomesodermin^+^ Th frequencies. In summary, Eomesodermin^+^ Th cells may shape a dysregulated and proinflammatory immune milieu, driving disease progression and neurodegeneration in secondary progressive multiple sclerosis with potential as both biomarker and therapeutic target.

## Teaser

Bongert *et al*. report that elevated frequencies of Eomes-expressing T helper cells in peripheral blood are associated with disease progression and infratentorial brain atrophy in secondary progressive multiple sclerosis. These findings suggest a role for Eomes^+^ T helper cells as a potential biomarker and therapeutic target in progressive disease.

## Graphical Abstract

**Graphical Abstract:**
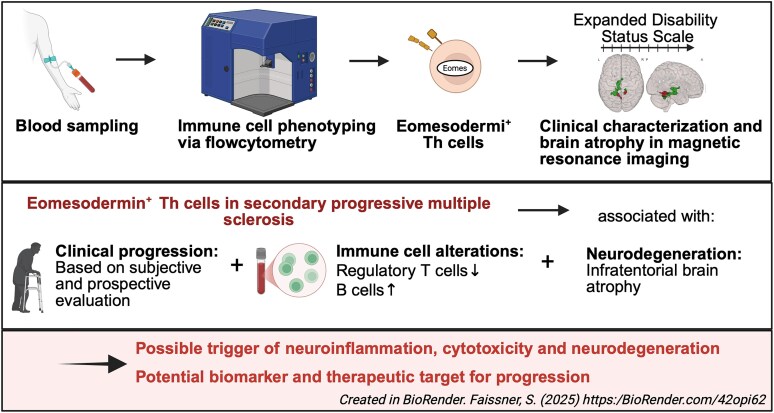

## Introduction

Multiple sclerosis (MS) is a chronic inflammatory disease of the central nervous system (CNS) characterized by demyelination, axonal damage and neurodegeneration.^1^ Due to the development of nearly 20 immunomodulating therapies effective for the relapsing-remitting phase of the disease (RRMS),^2,3^ both the risk of relapses and the risk of converting to a secondary-progressive disease course (SPMS) can be reduced substantially.^4^ Nevertheless, a substantial number of patients have underlying progression independent of relapse activity (PIRA); a phenomenon that received increasing attention during the last couple of years. Pathomechanisms fuelling progression include chronic inflammation behind an intact blood brain-barrier mediated by cells of innate immunity such as microglia, oxidative stress leading to mitochondrial damage and neurodegeneration as well as the activation of B cells and T cells.^5^ Targeting progressive MS remains difficult due to the complexity of the mechanisms involved. Early identification of underlying progression is an important goal to optimize treatment, for example, with highly effective disease-modifying therapies (DMTs). Well-established markers for neurodegeneration in MS are global brain atrophy in magnetic resonance imaging, retinal atrophy in optical coherence tomography and the release of neurofilament light chain in serum. Those parameters correlate with the clinical performance and progression in patients with MS.^6-8^ Recently, CD4^+^ T helper cells expressing the transcription factor Eomesodermin (Eomes^+^ Th cells) have been identified as a potential biomarker of progression.^9,10^ Eomes^+^ Th cells are upregulated during the chronic phase of experimental autoimmune encephalomyelitis (EAE), a mouse model for MS, and depict a cytotoxic phenotype through Granzyme B expression.^9^ Higher frequencies of Eomes^+^ Th cells are associated with active progression in SPMS and brain autopsy from a patient with SPMS revealed that Eomes^+^ Th cells expressing Granzyme B infiltrate the brain tissue.^10^ Those findings led to the assumption that Eomes^+^ Th cells could be a putative biomarker for progression in MS and play an important role in the pathogenesis of progressive MS.^10^ We here performed a prospective, longitudinal study and investigated the role of Eomes^+^ T cell subsets for progression in patients with MS and in healthy controls. We investigated whether patients with higher frequencies of Eomes^+^ Th cells might be at risk to progress longitudinally over one year and whether Eomes^+^ Th cells are associated with brain atrophy patterns measured by MRI.

## Materials and methods

### Standard protocol approvals, registrations and patient consents

The prospective and longitudinal study was approved by the Ethics Committee of the Faculty of Medicine of the Ruhr-University Bochum (Register-number: 20-6827) on 2 March 2020 and conducted in accordance with the Declaration of Helsinki. All patients and controls provided written informed consent.

### Enrolment of patients and healthy controls

Patients were enrolled at the Department of Neurology, Ruhr-University Bochum, St. Josef-Hospital. Included patients had a definite diagnosis of MS according to McDonald Criteria 2017.^11^ SPMS diagnosis required a continuous worsening of disease activity for more than six months with a first diagnosis of RRMS. Diagnosis of PPMS required a disease progression for more than one year without occurring relapses. All patients and controls had no competing diagnoses such as neoplasms or acute inflammatory diseases. Participants in the healthy control group were not allowed to have a malignant disease or an immunological disease including psoriasis or type 1 diabetes. Immunomodulatory therapy was also not allowed. All healthy controls (HC) were non-smokers and reported low or no alcohol use. Two patients retrospectively did not fulfil criteria for definite MS and were therefore excluded from the analysis.

### Clinical parameters

Neurological examination was performed by an experienced consultant neurologist. Demographic and disease-specific data such as disease duration, time to conversion to SPMS or recent immunomodulating therapies were collected from all patients enrolled in the study (Table 1). Information about current immunomodulating therapy is shown in Table 2. Expanded disability score (EDSS) and multiple sclerosis functional composite (MSFC) were analysed. Patients with a diagnosis of RRMS were subdivided according to their current clinical state. ‘At relapse’ was defined as neurologic deterioration for more than 24 h as a consequence of an acute demyelinating event. Patients ‘in remission’ were clinically stable for more than 30 days. Progressive patients were classified by deterioration of MSFC and EDSS within 12 months. Disease activity was defined as ‘active progressive’ with a deterioration of MSFC >20% or EDSS >0.5 and as ‘stable’ with a deterioration of MSFC <20% or EDSS <0.5.^12^ Subjective disease activity was obtained by asking patients using a survey if they retrospectively had the impression that they had been stable or progressing during the last year. Progressive patients with a subjective decline of disability in the last 12 months were characterized as ‘subjective progressive’. Biomarker analysis and clinical analysis were performed by different investigators blinded to their respective data. Number of cells and concentration of protein in cerebrospinal fluid were routinely investigated.

**Table 1:** Demographic and clinical data from patients and healthy controls (Baseline and Follow-up)

	HC Baseline	HC Follow-up	RRMS Baseline	RRMS Follow-up	SPMS Baseline	SPMS Follow-up	PPMS Baseline	PPMS Follow-up
Number	45	36	33	10	79	51	36	24
Male (%)/Female (%)	19 (42%)/26 (58%)	18 (50%)/18 (50%)	5 (15.2%)/28 (84.8%)	2 (20%)/8 (80%)	30 (38%)/49 (62%)	19 (37%)/32 (63%)	17 (47%)/19 (53%)	11 (46%)/13 (54%)
Age (mean) ± SD (range)	42.82 ± 15.83 (19–62)	43.3 ± 15.45 (20–63)	32.9 ± 8.61 (18–53)	39 ± 10 (19–53)	56.88 ± 10.52 (28–81)	59.29 ± 10.28 (28–82)	58.97 ± 9.79 (38–77)	59.42 ± 11.2 (39–78)
Disease duration (mean) ± SD (range)	—	—	6.0 ± 7.1 (0–29)	8.1± 5.4 (1–16)	23.0 ± 11.66 (3–60)	21.57 ± 9.98 (3–42)	13.87 ± 10.69 (0–41)	11.63 ± 9.12 (1–32)
EDSS (mean) ± SD (range)	—	—	2.37 ± 1.23 (1.0–6.0)	2.3 ± 0.86 (1.0–3.5)	5.6 ± 1.52 (2.0–8.0)	5.64 ± 1.5 (2.0–8.0)	4.98 ± 1.91 (1.0–8.0)	5.1 ± 1.85 (1.5–7.0)
EDSS (median)	—	—	2.5	2.5	6.0	6.0	6.0	6.0
EDSS ± SEM	—	—	2.37 ± 0.28	2.3 ± 0.3	5.6 ± 0.18	5.64 ± 0.2	4.98 ± 0.32	5.1 ± 0.4
Current relapse (%)	—	—	10 (30.3%)	1 (9%)	—	—	—	—

**Table 2:** Immunomodulating therapies

	RRMS (total 33)	RRMS FU (total 10)	SPMS (total 79)	SPMS FU (total 51)	PPMS (total 36)	PPMS FU (total 24)
No therapy	8 (23%)	1	18 (24%)	8	9 (25%)	2
B cell depletion	7 (20%)	3	32 (40%)	27	24 (67.5%)	21
Rituximab intrathecally	—		2 (2.5%)	2	—	—
S1P-modulators	3 (9%)	1	8 (10%)	4	—	—
Cladribine	3 (9%)		3 (4%)	1	—	—
Dimethyl fumarate	6 (18%)	3	9 (11%)	5	2 (5%)	1
Teriflunomide	—	—	1 (1%)	1	1 (2.5%)	—
Glatiramer acetate	3 (9%)	2	2 (2.5%)	2	—	—
Interferon beta-1a	1 (3%)	—	3 (4%)	1	—	—
Natalizumab	1 (3%)	—	1 (1%)	—	—	—
Alemtuzumab	1 (3%)	—	—	—	—	—

### Blood collection and PBMC isolation

Blood was collected before a cycle of intravenous administration of drugs such as glucocorticoids or anti-CD20 therapies. PBMCs were isolated as previously described.^13^ Cerebrospinal fluid was collected according to clinical practice guidelines and was stored at −80°C.

### Flow cytometry

Freshly isolated PBMCs were incubated with fluorochrome-conjugated antibodies as described^14^ (Supplementary Methods; Supplementary Table 1). Gating strategy is shown in Supplementary Fig. 1. Representative dot plots from flow cytometry analysis are shown in Fig. 2. We performed both isotype staining controls (Supplementary Table 2) as well as fluorescence minus one controls to distinguish Eomes⁺ from Eomes^−^ populations and to set gates that avoid false-positive inclusion (Supplementary Figs 3 and 4).

### MRI

In a subgroup of 41 MS patients, we exploratively performed a groupwise volumetric brain analysis based on 1.5 T MRI, comparing patients with higher and lower frequencies of Eomes^+^ Th cells (>2 versus <2) using the voxel-based morphometry (VBM) method processed on Statistical Parametric Mapping software (SPM12). MRI sequence and methodological details are provided in the Supplementary Materials. Due to the explorative character, we obtained significant clusters of brain volume reduction without correction of family-wise error for a *P*-value < 0.001 and an extent threshold of 50 voxels was set. Group comparisons between patients with frequencies of Eomes^+^ Th cells >2 and <2 were performed for grey and white matter volumes, building a statistical model based on a two-sample *t*-test using total intracranial volume and sex as covariates.

### Statistical analysis

Data were analysed with Prism software V.9.2.0 (GraphPad Software, San Diego, CA, USA). Normality test (Shapiro–Wilk test) was applied for all data sets. Comparison of two groups with non-parametric data was performed with Mann–Whitney U test. More than two non-parametric data sets were compared using Kruskal–Wallis test with Dunn’s multiple comparison. Data are presented as mean values with SD. Correlation was measured by non-parametric Spearman correlation and depiction of linear regression. Multivariate analysis was performed using R (Version 4.3.2) with a general linear model (GLM) (Supplementary Code 1). Depending on the distribution of the data, a Gaussian GLM model was chosen for normally distributed data and a gamma GLM for non-normally distributed data, as this model is particularly suitable for skewed data. A *P*-value < 0.05 was considered as statistically significant. As this was an exploratory observational study, the sample size was based on feasibility and available patient numbers during the recruitment period.

### Ethics approval and consent to participate

The study was approved by the local ethics committee of the Faculty of Medicine, Ruhr-University Bochum (Register-number: 20-6827) on 2 March 2020. All patients provided written informed consent.

### Consent for publication

All patients provided written consent for publication.

## Results

### Study population

**Figure 1:**
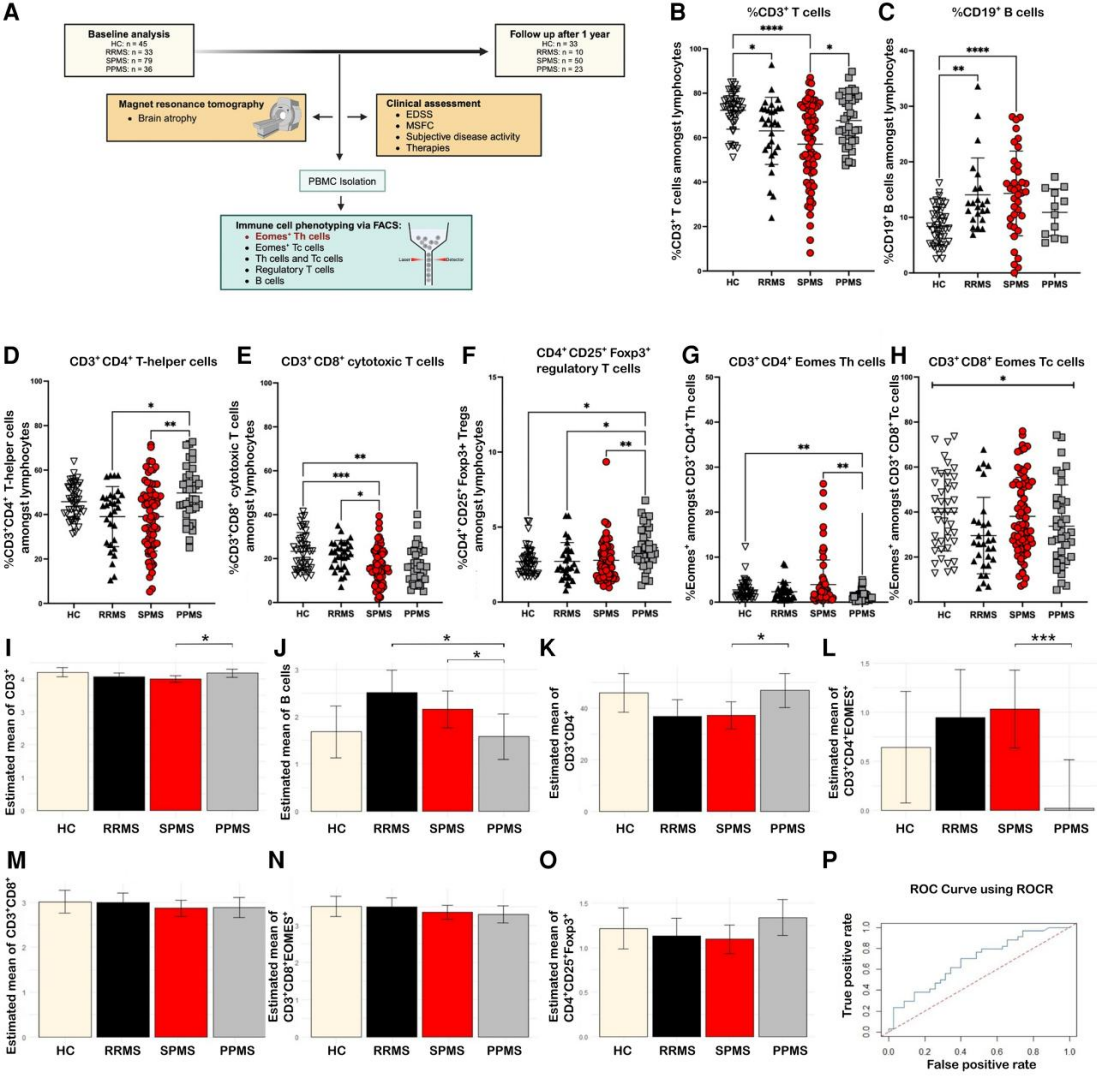
**Study protocol and immune cell subsets in patients with multiple sclerosis (baseline).** (**A**) Patients were characterized at baseline and 12 months after written informed consent. Clinical assessment and MRI parameters were obtained. Blood was drawn in order to analyse immune cell subsets by using flow cytometry. Created in BioRender. Faissner, S. (2025) https://BioRender.com/fqj3ayf. (**B**) CD3^+^ T cells were lower in RRMS patients and SPMS patients compared to healthy controls, while PPMS patients had higher CD3^+^ T cells compared to SPMS. (**C**) B cells were elevated in RRMS patients and SPMS patients compared to healthy controls. (**D**) CD3^+^ CD4^+^ Th cells were elevated in PPMS patients compared to SPMS patients and RRMS patients. (**E**) CD3^+^ CD8^+^ Tc cells were lower in SPMS patients and PPMS patients. (**F**) CD4^+^ CD25^+^ Foxp3^+^ regulatory T cells were elevated in PPMS patients compared to healthy controls, RRMS patients and SPMS patients. (**G**) Eomes^+^ Th cells were higher in SPMS patients compared to PPMS patients. PPMS patients had lower frequencies of Eomes^+^ Th cells than healthy controls. (**H**) Proportions of Eomes^+^ Tc cells did not differ clearly between groups. (**B–H)** Patients receiving S1P-modulators were excluded from data analysis. (**B–O**) Data are derived from *n* = 43 HC, *n* = 30 RRMS patients, *n* = 71 SPMS patients and *n* = 36 PPMS patients. Individual data points represent the measurement of one sample of one patient. Shown is a mean ± SD for each patient group. Data were analysed using non-parametric Kruskal–Wallis test with *post hoc* Dunn’s multiple comparison test. (**I–P**) Multivariate analysis and ROC analysis: Multiple GLM were performed to investigate the differences between the patients’ groups and HC regarding the lymphocyte subsets. Sex, age and therapy were included as covariates to determine any influences on the effect. Depending on the distribution of the data we chose a GLM of the gauss or gamma family, respectively. Therefore, for non-normal distributed data (**I** and **J**; **L–O**), the log-transformed adjusted means are displayed. (**P**) ROC analysis with an AUC of 0.681, indicating the model’s moderate predictive accuracy. *P < 0.05, **P < 0.01, ***P < 0.001, ****P < 0.0001. Abbreviations: AUC, area under the curve; EDSS, expanded disability status scale; Eomes, Eomesodermin; GLM, general linear model; HC, healthy controls; MSFC, multiple sclerosis functional composite; PBMC, peripheral blood mononuclear cells; PPMS, primary progressive multiple sclerosis; RRMS, relapsing remitting multiple sclerosis; SPMS, secondary progressive multiple sclerosis.

We included *n* = 33 patients with RRMS, *n* = 79 patients with SPMS, *n* = 36 patients with PPMS and *n* = 45 healthy controls, matched regarding sex and age (mean ± SD: 42.8 ± 15.8) (Table 1, Fig. 1A). SPMS and PPMS patients had a longer disease duration (RRMS: 6.0 ± 7.1; SPMS: 23.0 ± 11.7; PPMS: 13.9 ± 10.7) and higher EDSS (RRMS: 2.4 ± 1.2; SPMS: 5.6 ± 1.5; PPMS: 5.0 ± 1.9) than RRMS patients (*P* < 0.0001). *n* = 10 RRMS patients (30.3%) had a relapse in the last 8 weeks. A proportion of 77% of RRMS patients, 76% of SPMS patients and 75% of PPMS patients received DMTs(Table 2). Patients with progressive MS were predominantly treated with anti-CD20 antibodies such as rituximab and ocrelizumab [SPMS: *n* = 32 (40%); PPMS: *n* = 24 (67.5%)]. *n* = 2 SPMS patients received rituximab applied intrathecally. Further monoclonal antibodies included natalizumab [RRMS: *n* = 1 (3%); SPMS: *n* = 1 (1%)] and alemtuzumab [RRMS: *n* = 1 (3%)]. S1P modulators were applied in *n* = 3 RRMS patients (9%) and *n* = 8 SPMS patients (10%). Cladribine was taken by *n* = 3 RRMS patients (9%) and *n* = 3 SPMS patients (4%). Further DMTs included dimethyl fumarate [RRMS: *n* = 6 (18%); SPMS: *n* = 9 (11%); PPMS: *n* = 2 (5%)], teriflunomide [SPMS: *n* = 1 (1%); PPMS: *n* = 1 (2.5%)], glatiramer acetate [RRMS: *n* = 3 (9%); SPMS: *n* = 2 (2.5%)] and interferon beta-1a [RRMS: *n* = 1 (3%); SPMS: *n* = 3 (4%)]. A proportion of 23% of RRMS patients, 24% of SPMS patients and 25% of PPMS patients were without DMT at the time of recruitment.

### Immune cell subsets in multiple sclerosis and expression of Eomes in T cells

We first analysed T cell immune cell alterations (Fig. 1; Supplementary Table 3). Frequencies of CD3^+^ T cells were lower in RRMS patients (*P* < 0.05) and SPMS patients (*P* < 0.0001) compared to healthy controls, while PPMS patients had higher frequencies of CD3^+^ T cells compared to SPMS patients (*P* < 0.05) (Fig. 1B). B cells were higher in SPMS patients and RRMS patients compared to healthy controls (HC: 8.4 ± 3.4; RRMS: 14.1 ± 6.6; SPMS: 14.3 ± 7.6; PPMS: 10.9 ± 4.2; Fig. 1C). PPMS patients had higher frequencies of CD4^+^ Th cells compared to RRMS (*P* < 0.05) and SPMS (*P* < 0.01) (Fig. 1D). CD8^+^ Tc cells were lower in SPMS (*P* < 0.001) and PPMS (*P* < 0.01) compared to healthy controls (Fig. 1E). T_reg_ cells were found with higher frequencies in PPMS patients compared to healthy controls (*P* < 0.05), RRMS (*P* < 0.05) and SPMS (*P* < 0.01) (Fig. 1F). Absolute cell counts are shown in Supplementary Fig. 5.

As Eomes^+^ Th cells are thought to promote neurodegeneration in SPMS patients,^10^ we next compared the expression of Eomes^+^ T cells amongst circulating lymphocytes in peripheral blood. Frequencies of Eomes^+^ Th cells were significantly higher in SPMS compared to PPMS patients (SPMS: 3.9 ± 5.5; PPMS: 1.4 ± 1.1; *P* < 0.01; Fig. 1G). PPMS patients expressed significantly lower frequencies of Eomes^+^ Th cells than HC (HC: 2.73 ± 2.18; *P* < 0.01; Fig. 1G). Eomes^+^ Tc cells differed on a group level between HC, RRMS patients, SPMS patients and PPMS patients, but not in a *post hoc* analysis between respective groups (HC: 39.9 ± 17.3; RRMS: 29.5 ± 17.0; SPMS: 38.1 ± 17.3; PPMS: 33.7 ± 18.4; Fig. 1H). We performed a multivariate analysis, taking the covariates age, sex, treatment and baseline EDSS into consideration. Frequencies of CD3^+^ T cells were lower in SPMS patients compared to healthy controls. B-cells were higher in SPMS patients in comparison to PPMS patients (Fig. 1J; *P* = 0.034). We did not exclude patients receiving B cell–depleting therapies, as our study reflects a real-world cohort. The majority of patients were seen during routine follow-ups in daily clinical practice and mostly presented for the continuation of their infusion with B-cell depleting therapy. Importantly, blood sampling was performed prior to the next scheduled infusion, at a time when B cells were already repopulated, especially in rituximab-treated patients. CD4^+^ Th cells were higher in PPMS compared to RRMS and SPMS (Fig. 1D). Eomes^+^ Th cells as potential driver of neurodegeneration in SPMS patients were still significantly more abundant in SPMS compared to PPMS (Fig. 1L; *P* = 0.034 RRMS versus PPMS, *P* < 0.001 SPMS versus PPMS). CD8^+^ Tc cells did not differ. Regulatory T cells were higher in PPMS patients and HC in comparison to SPMS (Fig. 1O; *P* = 0.006 SPMS versus PPMS, *P* = 0.006 SPMS versus HC). A ROC analysis documented an area under the curve of 0.681 (Fig. 1P) indicating a moderate predictive performance. Hence, the multivariate analysis supported the main findings about the differentiation of frequencies of Eomes^+^ Th cells between SPMS and PPMS.

Eomes^+^ Th cells correlated with Eomes^+^ Tc cells, most prominent in RRMS patients (*R*^2^ = 0.51, *r* = 0.69, *P* < 0.0001) and SPMS patients (*R*^2^ = 0.22, *r* = 0.63, *P* < 0.0001; Supplementary Fig. 6). We observed no influence of age or sex on the frequencies of Eomes^+^ Th cells (Supplementary Fig. 7). However, Eomes^+^ Tc cells correlated with age in SPMS patients (*R*^2^ = 0.04, *r* = 0.23; *P* < 0.05) and PPMS patients (*R*^2^ = 0.17, *r* = 0.45; *P* < 0.01; Supplementary Fig. 8). We analysed whether the type of DMT has an influence on the frequencies of Eomes^+^ T cells. Most DMTs did not affect the frequencies of Eomes^+^ Th cells (Supplementary Fig. 9). On a group level, there was also no difference between treated and untreated patients (Supplementary Fig. 9D). However, S1P-modulators enhanced the percentual expression of Eomes by reducing the number of peripheral circulating T cells. By excluding patients treated with S1P modulating therapy in our analysis, we ruled out this bias provoked through S1P-modulators. Eomes^+^ Tc cells were not clearly altered through immunomodulation in RRMS, SPMS or PPMS patients (Supplementary Fig. 9E–G). Furthermore, we analysed changes of regulatory T cells through immunomodulation (Supplementary Fig. 9H–J). Interestingly, T_reg_ cells were significantly lower in SPMS patients treated with S1P-modulators compared to B-cell-depleting therapies (*P* < 0.05) and interferon beta-1a (*P* < 0.05). As T_reg_ cells were measured as a percentual proportion of total lymphocytes, the effect of S1P-modulators on T_reg_ cells can be considered as unbiased.

### Eomes^+^ Th cells are associated with lower regulatory T cells in SPMS patients and higher B cells

**Figure 2:**
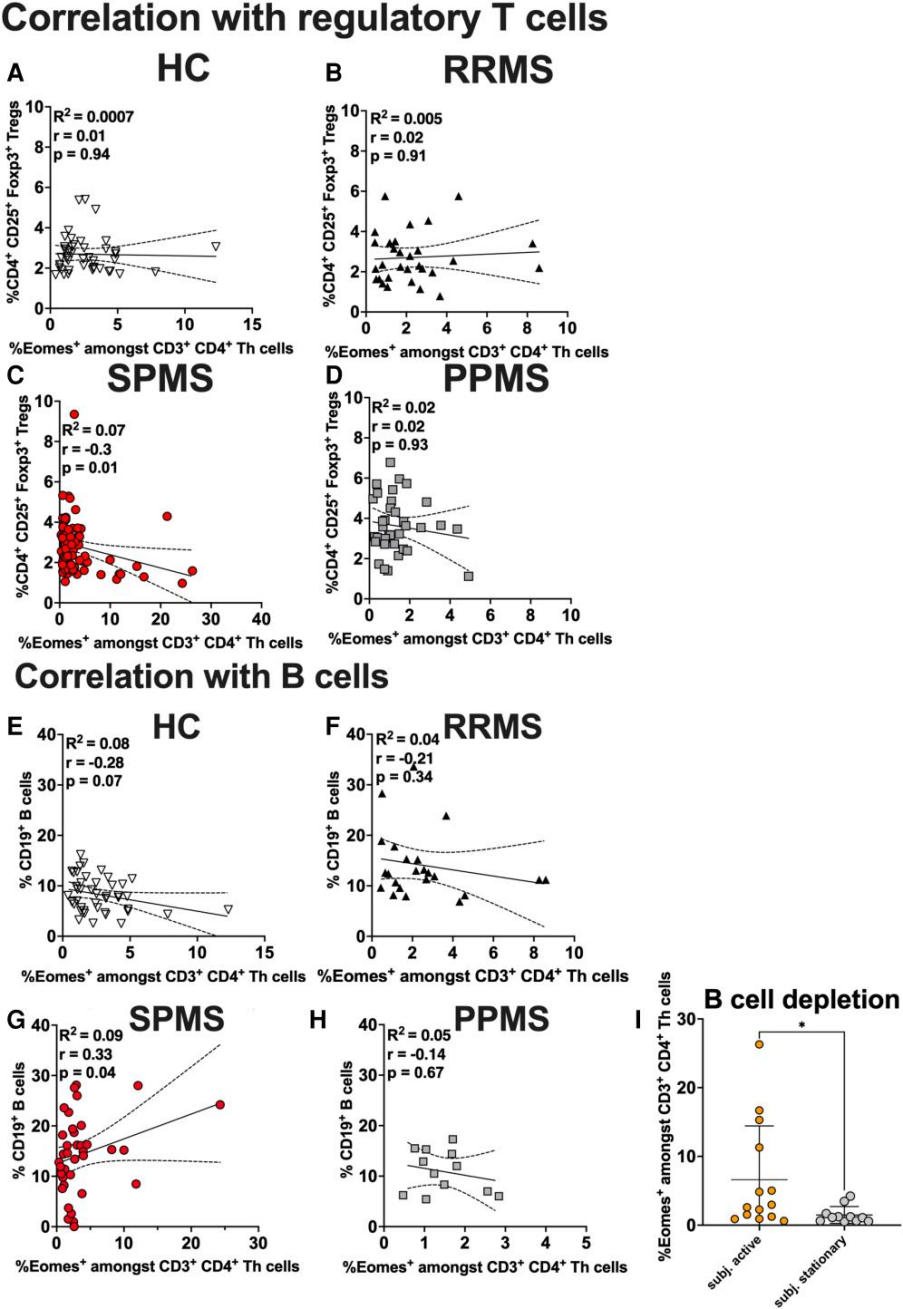
**Eomes^+^ Th cells are associated with lower frequencies of regulatory T cells and higher frequencies of B cells in SPMS patients.** Eomes^+^ Th cells did not correlate with the number of regulatory T cells in (**A**) HC (*r* = 0.01; *P* = 0.94), (**B**) RRMS patients (*r* = 0.02; *P* = 0.91) and (**D**) PPMS patients (*r* = 0.02; *P* = 0.93). (**C**) Eomes^+^ Th cells are associated with the frequency of regulatory T cells in SPMS patients (*r* = −0.3; *P* = 0.01). (**B+C**) Patients receiving S1P-modulators were excluded from data analysis. Data were derived from *n* = 43 HC, *n* = 30 RRMS patients, *n* = 71 SPMS patients and *n* = 36 PPMS patients. B cells did not correlate with the expression of Eomes on Th cells in (**E**) HC (*r* = −0.28; *P* = 0.07), (**F**) RRMS patients (*r* = −0.21; *P* = 0.34) and (**H**) PPMS patients (*r* = −0.14; *P* = 0.67). (**G**) However, higher frequencies of B cells correlate with the number of Eomes^+^ Th cells in SPMS patients (*r* = 0.33; *P* = 0.04). B cell depleted patients with a subjective decline of disease activity revealed higher Eomes+ Th cells (*P* = 0.02; *n* = 14 active, *n* = 11 stationary). (**E–H**) Patients receiving B cell-depleting therapies were excluded from data analysis. Data were derived from *n* = 43 HC, *n* = 24 RRMS, *n* = 37 SPMS and *n* = 17 PPMS. Individual data points represent the measurement of one sample of one patient. Shown is (**A–H**) linear regression with 95% confidence interval and (**I**) mean ± SD for each patient group. Data were analysed using (**A–H**) non-parametric Spearman test and (**I**) non-parametric Mann–Whitney Test. *P < 0.05. Abbreviations: Eomes, Eomesodermin; HC, healthy controls; PPMS, primary progressive multiple sclerosis; RRMS, relapsing remitting multiple sclerosis; SPMS, secondary progressive multiple sclerosis; Subj, subjectively.

Eomes^+^ Th cells are capable of restricting the peripheral Foxp3^+^ expression in regulatory T cells *in vitro*.^15^ HC, RRMS and PPMS showed no correlation between Eomes^+^ Th cells and T_reg_ cells (Fig. 2). Interestingly, we here observed a negative correlation of frequencies of regulatory T cells in peripheral blood with increasing frequencies of Eomes^+^ Th cells. This effect occurred exclusively in SPMS patients (*R*^2^ = 0.07; *r* = −0.3; *P* < 0.05; Fig. 2C). There was no common clinical or treatment pattern between patients with high frequencies of Eomes^+^ Th cells and low frequencies of CD25^+^FOXP3^+^ cells. Contrarily, frequencies of Eomes^+^ Tc cells did not correlate with frequencies of circulating T_reg_ cells (Supplementary Fig. 10).

As B cells play an important role during MS progression by enhancing inflammation and neurodegeneration, we analysed an interaction between Eomes^+^ Th cells and B cells. We only analysed patients who did not receive B cell depleting therapy in the past. Higher frequencies of B cells were associated with increased frequencies of Eomes^+^ Th cells in SPMS patients (*R*^2^ = 0.09, *r* = 0.33, *P* < 0.05); Fig. 2G). This correlation was not evident in other groups (Fig. 2). Of note, B cell-depleted patients with a subjectively active disease progression revealed higher frequencies of Eomes^+^ Th cells (*P* < 0.05; Fig. 2I) and lower frequencies of regulatory T cells (Supplementary Fig. 11). Other immunomodulating therapies were not associated with elevated Eomes^+^ Th cells in patients with a subjective disease progression (Supplementary Fig. 11).

### Eomes^+^ Th cells correlate with subjective clinical progression in SPMS

**Figure 3:**
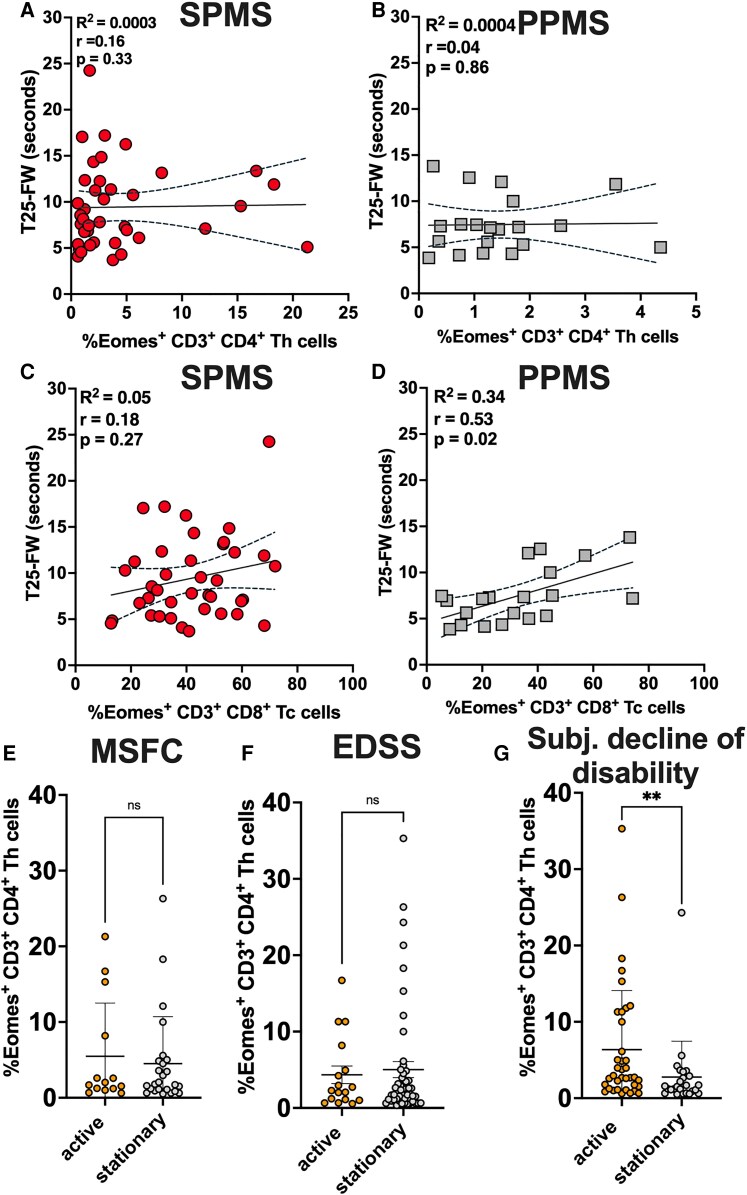
**Eomes^+^ Th cells and clinical progression (MSFC, EDSS, subjective). (A** and **B**) Time-25 Foot Walk did not correlate with Eomes^+^ Th cells in SPMS (*r* = 0.16; *P* = 0.33) and PPMS (*r* = 0.04; *P* = 0.86). (**C**) Time-25 Foot Walk did not correlate with Eomes^+^ Tc cells in SPMS (*r* = 0.18; *P* = 0.27). However, Eomes^+^ Tc cells showed a positive correlation with the Time-25 Foot Walk in PPMS patients (*r* = 0.53; *P* = 0.02). (**E–G**) SPMS patients were characterized as being from active or stationary status, based on deterioration in (**E**) MSFC or (**F**) EDSS. There was no difference between active or stationary patients regarding Eomes^+^ Th cells. (**G**) SPMS were characterized as being from progressive or stationary status, based on subjective decline of the disability. The survey of the current disease activity was conducted in a blinded manner. Subjectively progressive patients had higher number of Eomes^+^ Th cells (*P* = 0.007). Data were derived from **A**; (**C**) *n* = 39 SPMS patients, **B**; (**D**) *n* = 20 PPMS patients, (**E**) *n* = 14 active SPMS patients, *n* = 25 stationary SPMS patients; (**F**) *n* = 17 active SPMS patients, *n* = 51 stationary SPMS patients; (**G**) *n* = 37 active SPMS patients, *n* = 25 stationary SPMS patients. Individual data points represent the measurement of one sample of one patient. (**A–D**) Shown is a linear regression with 95% confidence interval and (**E** and **F**) mean ± SD for each patient group. Data were analysed using (**A–D**) non-parametric Spearman test and (**E** and **F**) non-parametric Mann–Whitney Test. Abbreviations: EDSS; expanded disability status scale; Eomes, Eomesodermin; MSFC, multiple sclerosis functional composite; PPMS, primary progressive multiple sclerosis; SPMS, secondary progressive multiple sclerosis; T25-FW; Time-25 Foot Walk.

To understand whether Eomes^+^ T cells might be associated with clinical worsening and transition to SPMS, clinical status in association with Eomes^+^ T cells was analysed both retrospectively in a cross-sectional analysis as well as prospectively over one year. First, neither Eomes^+^ Th cells nor frequencies of regulatory T cells correlated with the time to conversion to SPMS (Supplementary Fig. 12). Next, we investigated if Eomes^+^ Th cells are associated with progression in MS using different endpoints. The annual change of EDSS score ≥0.5 or change in MSFC ≥20% was considered as active progression. Retrospectively, there was no association between the frequencies of Eomes^+^ Th cells and clinical progression in the last 12 months (Fig. 3E and F). However, SPMS patients experiencing a subjective worsening of disease activity showed higher frequencies of Eomes^+^ Th cells in peripheral blood than patients being subjectively in a stationary state of the disease (active: 6.4 ± 7.8; stationary: 2.8 ± 4.7; *P* < 0.01; Fig. 3G). The survey of the current disease activity was conducted independently from flow cytometric measurements.

### Infratentorial brain atrophy in patients with higher frequencies of Eomes^+^ Th cells

**Figure 4:**
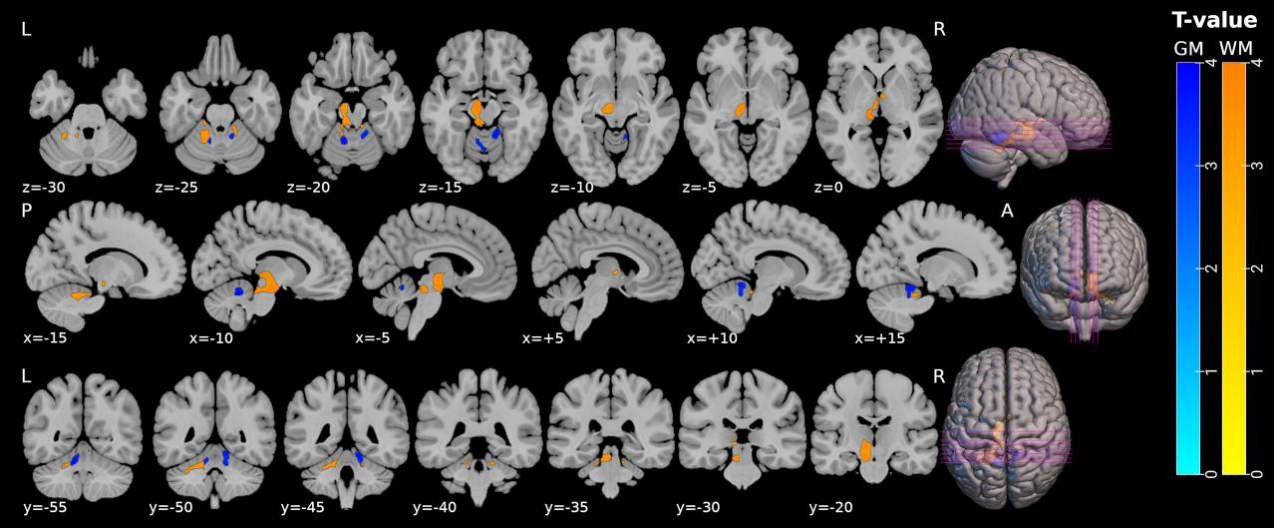
**MRI-based volumetric group comparison demonstrating pronounced infratentorial brain atrophy in patients with higher frequencies of Eomes^+^ Th cells compared to patients with lower frequencies.** Significant reduced clusters of white (colored in green) and grey (colored in red) matter volumes in patients with higher (>2) frequencies of Eomes^+^ Th cells (*n* = 19) compared to patients with lower frequencies (<2) (*n* = 20). Upper row: axial slices; middle row: sagital slices; lower row: coronal slices. Image orientation: L = left, R = right, *P* = posterior, A = anterior. Slice position in MNI space is indicated by the *x*/*y*/*z* coordinates below the images. Abbreviations: Eomes, Eomesodermin; GM, grey matter; WM, white matter. Details of statistical parameter mapping are provided in the Supplementary Materials.

VBM analysis comparing patients with frequencies of Eomes^+^ Th cells >2 (*n* = 19) and <2 (*n* = 22) revealed contiguous clusters of significantly reduced white and grey matter volumes in infratentorial (midbrain and cerebellar) regions in patients with higher (>2) frequencies of Eomes^+^ Th cells. Testing the counterhypothesis showed no significant results. Anatomical locations of significant clusters for cross-sectional group comparisons determined by atlas-based extraction are provided in Supplementary Table 4. Figure 4 shows the significantly reduced regions of white and grey matter volumes in patients with frequencies of Eomes^+^ Th cells >2 compared to <2.

### Frequencies of baseline Eomes^+^ Th cells correlate with disability progression after one year

**Figure 5:**
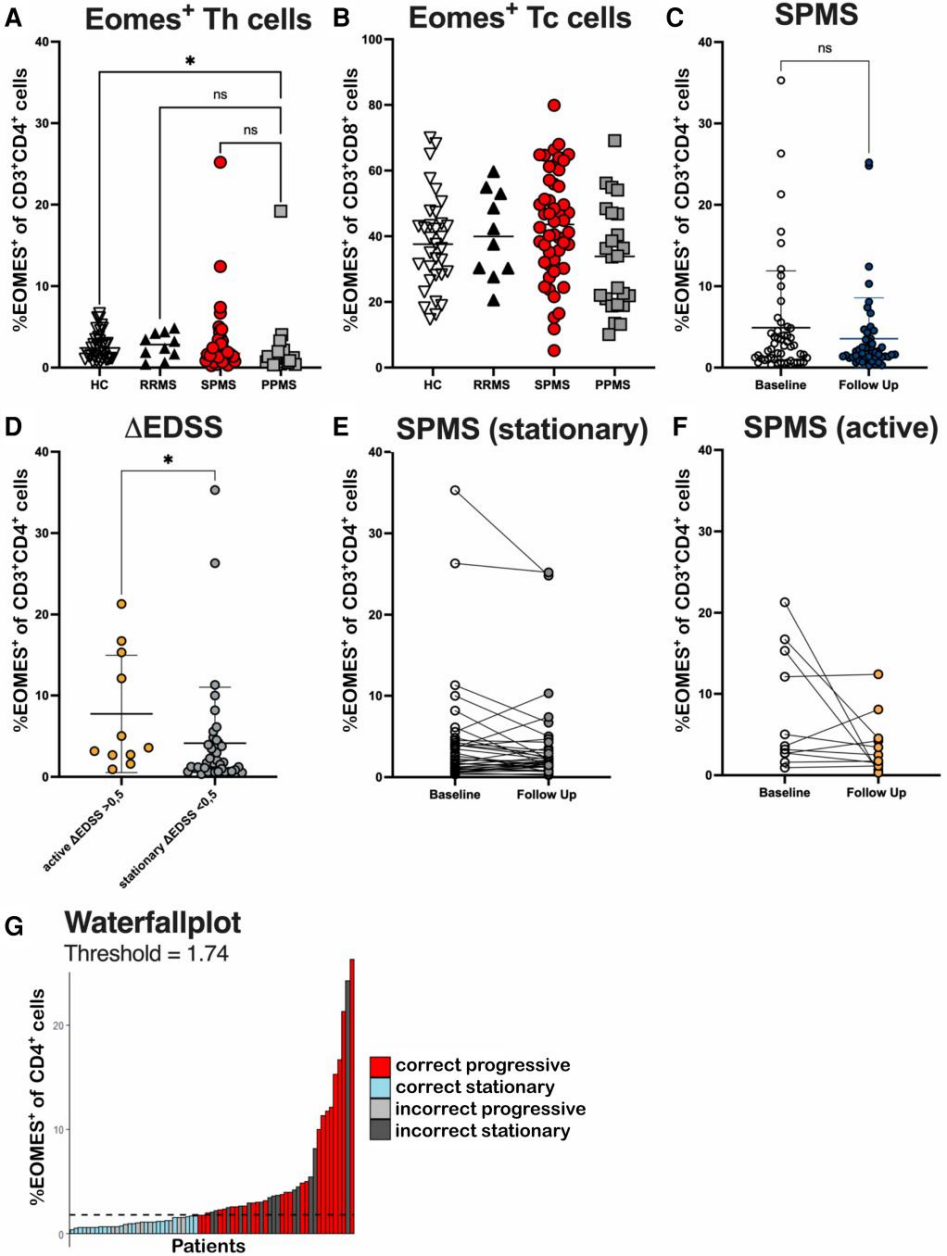
**Patients with higher frequencies of Eomes+ Th cells show EDSS progression after one year.** (**A**) Frequencies of Eomes^+^ Th cells were lower in PPMS patients compared to HC. (**B**) Frequencies of Eomes^+^ Tc cells did not differ between groups at follow-up. (**C**) Eomes^+^ Th cells in SPMS patients were not altered between baseline and follow-up after 12 months. (**D**) SPMS patients with prospective disease progression after 12 months measured using the EDSS had higher frequencies of Eomes^+^ Th cells at baseline. (**E**) Eomes^+^ Th cells in stationary SPMS patients did not significantly change between baseline and follow-up. (**F**) Eomes^+^ Th cells in active SPMS patients did not change between baseline and follow-up. (**G**) Optimal cut-off for Eomes^+^ Th cells (1.74%, Youden Index) effectively distinguishes stationary and progressive disease (waterfall plot). (**A** and **B**) Patients receiving S1P-modulators were excluded from data analysis. Data were derived from (**A** and **B**) *n* = 35 HC, *n* = 10 RRMS, *n* = 45 SPMS, *n* = 24 PPMS, (**C**) *n* = 51 SPMS. (**D–F**) *n* = 11 active SPMS patients, *n* = 39 stationary SPMS patients. Characterization based on ΔEDSS < 0.5 = active, ΔEDSS>0.5=stationary. Individual data points represent the measurement of one sample of one patient. (**A+B**) Shown is a mean for each patient group. (**C+D**) Shown is a mean ± SD for each group. Data were analysed using (**A+B**) non-parametric Kruskal–Wallis test with *post hoc* Dunn’s multiple comparison test. *P < 0.05, **P < 0.01, ***P < 0.001. (**C**) non-parametric Wilcoxon Test. (**D**) non-parametric Mann–Whitney Test. Abbreviations: EDSS, expanded disability status scale; Eomes, Eomesodermin; HC, healthy controls; PPMS, primary progressive multiple sclerosis; RRMS, relapsing remitting multiple sclerosis; SPMS, secondary progressive multiple sclerosis; Subj, subjectively.

To understand both the dynamics of immune cell alterations and the association with clinical phenotype the cohort was followed up after one year. The follow-up cohort comprises a number of *n* = 10 RRMS patients, *n* = 51 SPMS patients and *n* = 24 PPMS patients. We collected a follow-up of *n* = 36 healthy controls (Table 1). EDSS was similar to the baseline (Table 1, RRMS: 2.3 ± 0.86, SPMS: 5.64 ± 1.5, PPMS: 5.1 ± 1.85). At follow-up, we observed no major changes in the frequencies of Eomes^+^ Th cells in the different groups compared to baseline. Frequencies of Eomes^+^ Th cells were lower in PPMS patients compared to HC (*P* = 0.01). Expression of Eomes in CD8^+^ Tc cells did not differ between groups (Fig. 5B; Supplementary Fig. 13). There were individual fluctuations of the frequencies of Eomes^+^ Th and Eomes^+^ Tc cells after one year in SPMS, RRMS and PPMS patients, but we did not see a significant change of Eomes^+^ Th cells after one year (Fig. 5C). Clinically, patients were followed regarding EDSS progression. This was defined as active (Delta EDSS >0.5) or stationary (Delta EDSS <0.5). There were *n* = 40 patients with stationary disease activity and *n* = 11 active SPMS patients. Patients with prospective active disease progression had significantly higher frequencies of Eomes^+^ Th cells at baseline compared to stationary patients (*P* = 0.03; Fig. 5D), supporting a role of Eomes^+^ Th cells for prospective progression in SPMS. There were no significant changes in Eomes^+^ Th cells in active or stationary SPMS patients between baseline and follow-up measurement (Fig. 5E and F). The optimal cut-off value for Eomes^+^ Th, calculated using the Youden Index, was determined to be 1.74%. A waterfall plot showed that most patients were correctly classified as having either stationary or progressive disease based on this cut-off (Fig. 5G).

## Discussion

Progression in multiple sclerosis is driven by complex immunological mechanisms. Predicting disease progression at an early stage remains still challenging. Here we showed that frequencies of Eomes^+^ Th cells are associated with subjective disease progression, prospective disability progression measured using EDSS after one year and more infratentorial, mainly cerebellar and midbrain, white and grey matter brain atrophy as a marker of neurodegeneration.

There has been increasing attention regarding cytotoxic T cells as potential drivers of MS progression. Eomes^+^ Th cells are upregulated in chronic lesions of EAE and are associated with chronic neuroinflammation and cytotoxic properties.^9,16^ Furthermore, microglia promote neurotoxic properties of Eomes^+^ Th cells during the neurodegeneration-associated phase of EAE.^17^ The blockage of microglia leads to reduced frequencies of Eomes^+^ Th cells in the CNS,^17^ suggesting an interaction of innate immune cells with Eomes^+^ Th cells.

We here showed that Eomes^+^ Th cells are elevated in SPMS patients while having a low expression in PPMS patients, which is in line with previous studies.^10^ While genome-wide association studies indicate shared genetic risks between secondary progressive and primary progressive multiple sclerosis, the differences in immune cell phenotypes, particularly the enrichment of Eomes-expressing T helper cells, could reflect differing inflammatory activity in SPMS and PPMS, not necessarily distinct pathogenic pathways. Indeed, Granzyme B-producing Eomes^+^ Th cells form a specific subset of T cells in SPMS that can induce neuroinflammation, cytotoxicity and disease progression, distinguishing between RRMS and SPMS with a sensitivity of 50%.^10^ In the cohort investigated here, the difference was only observable on a group level, presumably due to more conservative gating using flow cytometry. In the Japanese cohort investigated by Raveney *et al*., a higher expression of Eomes^+^ Th cells was associated with an active disease progression with increased neurological disability.^10^ This cohort was based on a cross-sectional analysis with a lower number of patients with repetitive samples (*n* = 16). In our longitudinal and prospective cohort, a larger number of patients was followed longitudinally with both immunological and clinical characterization. We here show for the first time that patients with higher frequencies of baseline Eomes^+^ Th cells are at risk of progression measured using the EDSS. We also demonstrated that Eomes^+^ Th cells were associated with lower frequencies of regulatory T cells only in SPMS patients. Foxp3, an important transcription factor of Treg cells, is suppressed by Eomes *in vitro.*^15^ While it remains unclear whether there is a direct effect of high frequencies of Eomes^+^ Th cells on regulatory T cells, those data suggest that Eomes^+^ Th cells possibly drive the creation of a dysbalanced and destabilized immune milieu in SPMS. Thus, the expression of Eomes could promote disease progression and increased neurodegeneration both through direct neurotoxic effects and also by restricting the regulatory properties of Treg cells. Furthermore, Eomes-expressing T-helper cells have also been described in the context of chronic viral infections, showing an exhaustion-like phenotype.^18^ Thus, Eomes^+^ Th cells may not only reflect active pro-inflammatory functions in SPMS but could also indicate a state of T cell exhaustion. However, Joulia *et al*. demonstrated recently that Eomes expression can actively drive chronic neuroinflammation by enhancing mitochondrial function and promoting resistance to apoptosis in T helper cells.^19^ These findings indicate, that Eomes^+^ Th cells might also act as active drivers of sustained neuroinflammatory responses in progressive MS. Furthermore, higher numbers of cells in the cerebrospinal fluid were associated with Eomes^+^ Th cells in SPMS patients, indicating an inflammatory phenotype of Eomes. As B cells correlated positively with Eomes^+^ Th cells exclusively in SPMS patients, those cells may form an inflammatory immune cell compartment in this subpopulation of patients. B cells are an important driver of disease progression in MS by establishing a proinflammatory status through the production of inflammatory cytokines,^20^ the activation of T cells^21,22^ and the formation of lymphoid follicular structures in the meninges.^23^ B cell targeting anti-CD20 antibodies such as rituximab, ocrelizumab, ofatumumab and ublituximab are an effective therapeutic approach in MS.^24-32^ Neither B cell depletion nor other immunomodulating therapies showed an effect on frequencies of Eomes^+^ Th cells in our study. Of note, B cell-depleted patients with a subjectively active disease progression showed higher frequencies of Eomes^+^ Th cells and lower frequencies of Treg cells. These patients may represent a subset of patients in whom B cell depletion is not effective in preventing disease activity, with Eomes^+^ Th cells driving disease progression instead. A putative reason for this might be activation through microglia, which are known to stimulate Eomes^+^ Th cells in the progressive phase of EAE.^17^

SPMS patients who experienced a subjective worsening of disease activity showed higher frequencies of Eomes^+^ Th cells in peripheral blood at baseline. Furthermore, we show a correlation between higher expression of Eomes in CD4^+^ Th cells in SPMS patients at the baseline measurement and clinical progression of the disease after one year, measured by changes in EDSS. These findings support the role of Eomes^+^ Th cells in driving secondary MS progression. Additionally, explorative brain volume analysis revealed a pronounced volume loss of infratentorial (mainly cerebellar and midbrain) white and grey matter in patients with higher frequencies of Eomes^+^ Th cells. Especially cerebellar atrophy occurs in progressive MS and is associated with disability,^33^ which possibly underlines the progressive disease character in patients with higher frequencies of Eomes^+^ Th cells in this context.

The concept of progression has evolved in recent years. Progression independent of relapse activity is considered to appear even at early stages of multiple sclerosis.^34^ Data from a pooled analysis of two randomized clinical trials (OPERA I and OPERA II) revealed that most disability accumulation in relapsing multiple sclerosis is independent of relapses.^35^ PIRA can be found in 25% of patients following a first demyelinating event and is associated with a steeper worsening of EDSS and long-term outcomes.^36^ Furthermore, patients with PIRA had a greater loss of brain volume especially in the cerebral cortex.^37^ Those findings challenge the clinically well-established differentiation between relapsing and progressive MS.^35^ While it is currently under debate which clinical markers should be used to best assess PIRA, the data shown here suggest that Eomes^+^ Th cells might be a substantial driver of disability. Indeed, a high level of perivascular inflammation, characterized by T and B cells, and active demyelination are associated with rapid progression in a post-mortem study.^38^ This is in line with findings from our group demonstrating that patients with pregnancy in MS showed a correlation of Eomes^+^ Th cells with reduced Treg cells and a significant increase of B-cells during the third trimester and a positive correlation with disability post-partum.^14^

Additionally, Eomes^+^ Th cells might serve as a therapeutic target for progressive MS, which should be investigated in the future. For instance, the cytokine TGF-β suppresses the expression of Eomesodermin in cytotoxic T cells in a mouse model.^39^ Furthermore, the treatment of mice with TGF-β ameliorates clinical symptoms of the EAE.^40^ However, a phase-1 trial of TGF-β2 in a small number of SPMS patients did not show an effect on EDSS or lesions in MRI but was associated with reversible nephrotoxicity.^41^

We here investigated a real-world cohort associated with different limitations such as intake of different DMTs with different modes of action impacting on immune cell composition. Moreover, we had a drop-out of patients at follow-up, which was, however, with 62% of the baseline population justifiable. Brain volume analysis has to be interpreted in an explorative context due to limited MRI data sets. Including healthy controls would be valuable, but since this analysis focused on differences between Eomes-high and Eomes-low patients, they were not included. This should be addressed in future studies.

We acknowledge that heteroscedasticity and the presence of potential outliers can affect the robustness and interpretability of correlation analyses. To minimize such effects, we used Spearman’s rank correlation, which is a non-parametric method that does not rely on distributional assumptions and is less sensitive to outliers. To further evaluate the potential impact of heteroscedasticity, we performed the Breusch–Pagan test on the residuals of the fitted linear regression models. For the significant correlations shown in Fig. 2G and C, the Breusch–Pagan statistics were 0.154 (*P* = 0.695) and 0.004 (*P* = 0.951), respectively. Both indicate that there is no significant evidence to reject the null hypothesis of homoskedasticity. These results suggest that the variance of the residuals is constant across fitted values and the assumption of homoskedasticity holds for our data. Additionally, visual inspection of residuals versus fitted values plots revealed no noticeable patterns. Nevertheless, we explicitly acknowledge the limitations associated with correlation analyses in complex immunological data and encourage further validation in larger, independent cohorts.

A major strength of the study consists of the inclusion of a large prospective and longitudinal cohort of patients with different disease courses with clinical, immunological and radiological phenotyping at a single university hospital setting without influence on immune cell measurements through different sites.

In summary, we provide evidence that Eomes^+^ Th cells are associated with prospective disability accumulation and infratentorial brain atrophy in MS. This might in part be driven by associated immune cell disbalances with increased frequencies of B cells and reduced frequencies of regulatory T cells. Future research should focus on the interaction of Eomes^+^ Th cells with innate immune cells and B cells.

## Supplementary Material

fcaf415_Supplementary_Data

## Data Availability

All data are available upon reasonable request. The script for the general linear model is provided in Supplementary Code 1.
