# Supplementary material for: Eomesodermin-expressing CD4 Th cells and association with multiple sclerosis progression and brain atrophy
Source: Brain Commun. 2025 Oct 27;7(6):fcaf415. doi: 10.1093/braincomms/fcaf415 (PMC12673330; doi:10.1093/braincomms/fcaf415)
Supplement: fcaf415_Supplementary_Data [file fcaf415_supplementary_data.zip › Supplementary Materials.pdf]

Eomesodermin expressing CD4 Th Cells and association with Multiple Sclerosis Progression and Brain Atrophy

SUPPLEMENTARY MATERIALS

SUPPLEMENTARY METHODS

Sex as a biological variable. Our study examined male and female patients. Sex was not considered as biological variable in the overall analysis, but in the multivariate analysis in Figure 1.

Flow Cytometry. Freshly isolated PBMCs were incubated with fluorochrome-conjugated antibodies against surface antigens (eBioscience™ and BioLegend®, Table 1) for 30 minutes at 4°C. Afterwards the cells were fixed and permeabilized with a Transcription Factor Staining Buffer Set (eBioscience™) according to the manufacturer's instructions. Cells were stained intracellularly with fluorochrome-conjugated antibodies against Eomes and Foxp3 (eBioscience™, Table 1/2) for 60 minutes at room temperature with continuous shaking (150 rpm). Flow cytometry data were recorded using a FACS Canto II (BD Biosciences, Franklin Lakes, NJ, USA). Analysis of the data was performed with FlowJo software V.10.7.2 (FlowJo, Becton, Dickinson and Company, Ashland OR, USA). Lymphocytes were gated according to forward and side scatter properties (Figure 1/2). T cells were identified by CD3 expression. T cell subsets were then selected by expression of CD4 or CD8. Percentual expression of Eomes in $CD3^+$ $CD4^+$ Th cells and in $CD3^+$ $CD8^+$ Tc cells was analysed. Regulatory T cells were identified as $CD4^+$ Th cells with simultaneous expression of CD25 and Foxp3. B cells were gated through $CD19^+$ expression.

Magnetic resonance imaging (MRI) analysis

Imaging was performed with a single 1.5Tesla (T) scanner (Siemens Avanto) using a standardized imaging protocol, with isotropic 3D fluid attenuated inversion recovery (FLAIR) sequence for lesion quantification (resolution 1 × 1 × 1 mm3, TR / TE / TI / FA : 6000 ms / 305 ms / 2100 ms/120°, acquisition time 7:30 min) and structural isotropic T1-weighted 3D sequence (resolution 1 × 1 × 1 mm^3, TR / TE / TI / FA : 1910 ms / 2,1 ms / 1100 ms/15°, acquisition time 5:14 min).

Assessment of $Eomes^+$ Th frequency cut-off

The choice of a cut-off of 2 was based on the intention to particularly map a difference in brain volumes between the relapsing and secondary progressive MS patients in relation to their frequencies of Eomes+ Th cells. Regarding the median distribution of $Eomes^+$ Th cells, RMS patients have a value of 1.885(0.97-2.68) and SPMS patients a value of 2.05(0.9-4.535). The

rationale for using a cut-off of 2 is based on this frequency distribution. Relapsing and secondary progressive MS patients for whom MRI data were available (n=41), were randomized irrespective to their disease subtype, into a group with higher frequency (>2; n=19) and a group with lower frequency (<2; n=22) based solely on their EOMES frequency in order to predominantly capture the difference based on the immune characteristic and not the declared disease subtype.

Volumetric Analysis

Volumetric and surface-based processing was performed using Statistical Parametric Mapping software (SPM 12, revision 7771) and Computational Anatomy Toolbox 12 (CAT12, version 12.8 (r1932), Structural Brain Imaging Group, University of Jena)[1] run with MATLAB version R2018b (Mathworks, Inc., Massachusetts, USA). Prior to volumetric analysis, lesion segmentation toolbox (LST toolbox, version 3.0.0) for SPM12 was used for lesion segmentation and filling in order to correct 3D-T1 weighted images for WM lesions.[2] Data preprocessing was performed using the default preprocessing steps of basic voxel-based morphometry analyses with CAT12 for normalizing, segmenting, and smoothing the data.[1] Gray and White matter images resulting from segmentation were smoothed with an 8mm full width half maximum (FWHM) Gaussian kernel and normalized with total intracranial volume (TIV).
Significant clusters were obtained without correction of family-wise error (FWE) for a p-value < 0.001 and an extent threshold of 50 voxels was set.
The anatomical locations of significant clusters in gray and white matter were determined with the SUIT[3] for cerebellar regions and Mori[4] atlas for cerebellar an additional midbrain regions. The cut-off for an overlap of the atlas was set to >=5%.

Supplementary Table 1: Antibodies used for flow cytometry.

Antigen	Conjugate	Clone	Ab-number	Supplier
CD3	PE-Cyanine7	UCHT1	AB_1582253	eBioscience
CD4	FITC	RPA-T4	AB_1659694	eBioscience
CD8	PE	RPA-T8	AB_314126	Biolegend
CD25	APC	BC96	AB_314280	Biolegend
CD19	PE-Cyanine7	HIB19	AB_1582278	eBioscience
Foxp3	PE	236A/E7	AB_1944444	eBioscience
Eomes	eFluor660	WD1928	AB_2574229	eBioscience

Supplementary Table 2: Isotype-controls.

Isotype	Conjugate	Clone	Ab-number	Supplier
Mouse IgG1 kappa	PE-Cyanine7	P3.6.2.8.1	AB_1548705	eBioscience
Mouse IgG1 kappa	APC	MOPC-21	AB_326443	Biolegend
Mouse IgG1 kappa	FITC	P3.6.2.8.1	AB_470022	eBioscience
Mouse IgG1 kappa	PE	MOPC-21	AB_2847829	Biolegend
Mouse IgG1 kappa	eFluor660	P3.6.2.8.1	AB_10597301	eBioscience

Supplementary Table 3: Descriptive statistics (mean, standard deviation (SD), standard error of the mean (SEM), and median) of immune cell subsets.

**Baseline**	**HC**	**RRMS**	**SPMS**	**PPMS**
**CD3$^+$ T cells**				
Mean + SD	72.32 + 8.5	63.05 + 15.1	57.06 + 18.3	67.7 + 11.5
SEM	1.3	2.6	2.2	1.9
Median	73.9	66.6	59.4	66.6
**CD4$^+$ Th cells**				
Mean + SD	45.8 + 7.9	39.1 + 13.5	39.1 + 15.5	49.7 + 12.4
SEM	1.2	2.5	1.8	2.1
Median	46	43.5	39.2	49.1
**CD8$^+$ Tc cells**				
Mean + SD	23.3 + 8.6	21.7 + 6.6	16.7 + 8.2	16.9 + 8.3
SEM	1.3	1.2	1	1.4
Median	23.5	22.7	15.3	22.7
**Treg cells**				
Mean + SD	2.7 + 0.9	2.7 + 1.3	2.8 + 1.3	3.6 + 1.4
SEM	0.1	0.2	0.2	0.2
Median	2.5	2.3	2.6	3.5
**Eomes$^+$ Th cells**				
Mean + SD	2.7 + 2.2	2.3 + 2	3.9 + 5.5	1.4 + 1.1
SEM	0.3	0.4	0.6	0.2
Median	2.3	1.9	2	1
**Eomes$^+$ Tc cells**				
Mean + SD	39.9 + 17.3	29.5 + 17	38.1 + 17.3	33.7 + 18.4
SEM	2.6	3.1	2.1	3.1
Median	40.2	26.2	34.4	30.2
**B cells**				
Mean + SD	8.4 + 3.4	14.1 + 6.6	14.3 + 7.6	10.9 + 4.2
SEM	0.5	1.4	1.3	1.2
Median	8	12.4	14.6	11.3
**Follow-up**				
**CD3$^+$ T cells**				
Mean + SD	73.11 + 8.8	67 + 21.1	65.2 + 17.2	78.2 + 7.6
SEM	1.5	6.7	2.4	1.5
Median	75.6	74.2	69	80.7
**CD4$^+$ Th cells**				
Mean + SD	45.8 + 8.1	44.1 + 18.7	44.3 + 16.6	59.2 + 10.8
SEM	1.4	5.9	2.3	2.2
Median	45.6	45.3	45.9	60.4
**CD8$^+$ Tc cells**				
Mean + SD	24.3 + 9.3	20.5 + 11.3	19.2 + 8.5	17.9 + 8.4
SEM	1.6	3.6	1.2	1.7
Median	24	25.3	19.1	17.1
**Treg cells**				
Mean + SD	2.9 + 0.7	3.5 + 1.3	3.8 + 1.6	4.7 + 1.4
SEM	0.1	0.4	0.2	0.3
Median	2.8	3.3	3.6	4.5
**Eomes$^+$ Th cells**				
Mean + SD	2.5 + 1.8	2.7 + 1.6	2.9 + 4.0	2.0 + 3.8
SEM	0.3	0.5	0.6	0.8
Median	1.7	2.8	1.7	1.1
**Eomes $^+$ Tc cells**				
Mean + SD	36.7 + 15.2	40.5 + 13.2	43.5 + 16.3	33.4 + 16.1
SEM	2.6	4.2	2.3	3.3

Median	36.3	40	44.5	33.9
**B cells**				
Mean + SD	7.8 + 4.1	6.7 + 5.4	5.5 + 7.6	2.6 + 3.5
SEM	0.7	1.7	1.1	0.7
Median	6.5	7.4	2	0.8

Abbreviations: Eomes, Eomesodermin; HC, Healthy controls; PPMS, primary progressive multiple sclerosis; RRMS, relapsing remitting multiple sclerosis; SPMS, secondary progressive multiple sclerosis; Treg cells, Regulatory T cells.

Supplementary Table 4: Significant VBM clusters for cross-sectional group comparisons between groups EOMES<2 and EOMES>2. Anatomical locations of significant clusters in GM and WM were determined with SUIT atlas, for WM the Mori (*) atlas was additionally used. The cut-off for an overlap of the atlas was set to 5%.

Anatomical region	Cluster size (voxels)	Overlap of atlas	T-value	p-value ($p_{FWE-corr}$)	(p_{uncorr})	MNI coordinates		
Gray matter								
**Left V**	223	85.00%	4	0,5944	0,1559	-10	-54	-22
**Left I IV**		7.00%						
**Right I IV**	269	45.00%	3.8	0,5002	0,1198	12	-46	-14
**Right V**		43.00%						
White matter								
**Right I IV**	85	73.00%	4.4	0,7388	0,3501	14	-36	-21
**Right V**		19.00%						
**Left V**	1557	12.00%	4.2	0,0046	0,0012	-8	-10	-18
**Left VI**		9.00%						
**Left I IV**		5.00%						
**Left Midbrain***		19.00%						
**Left Thalamus***		19.00%						
**Left Cerebral Peduncle***		15.00%						

*anatomical regions determined with Mori atlas

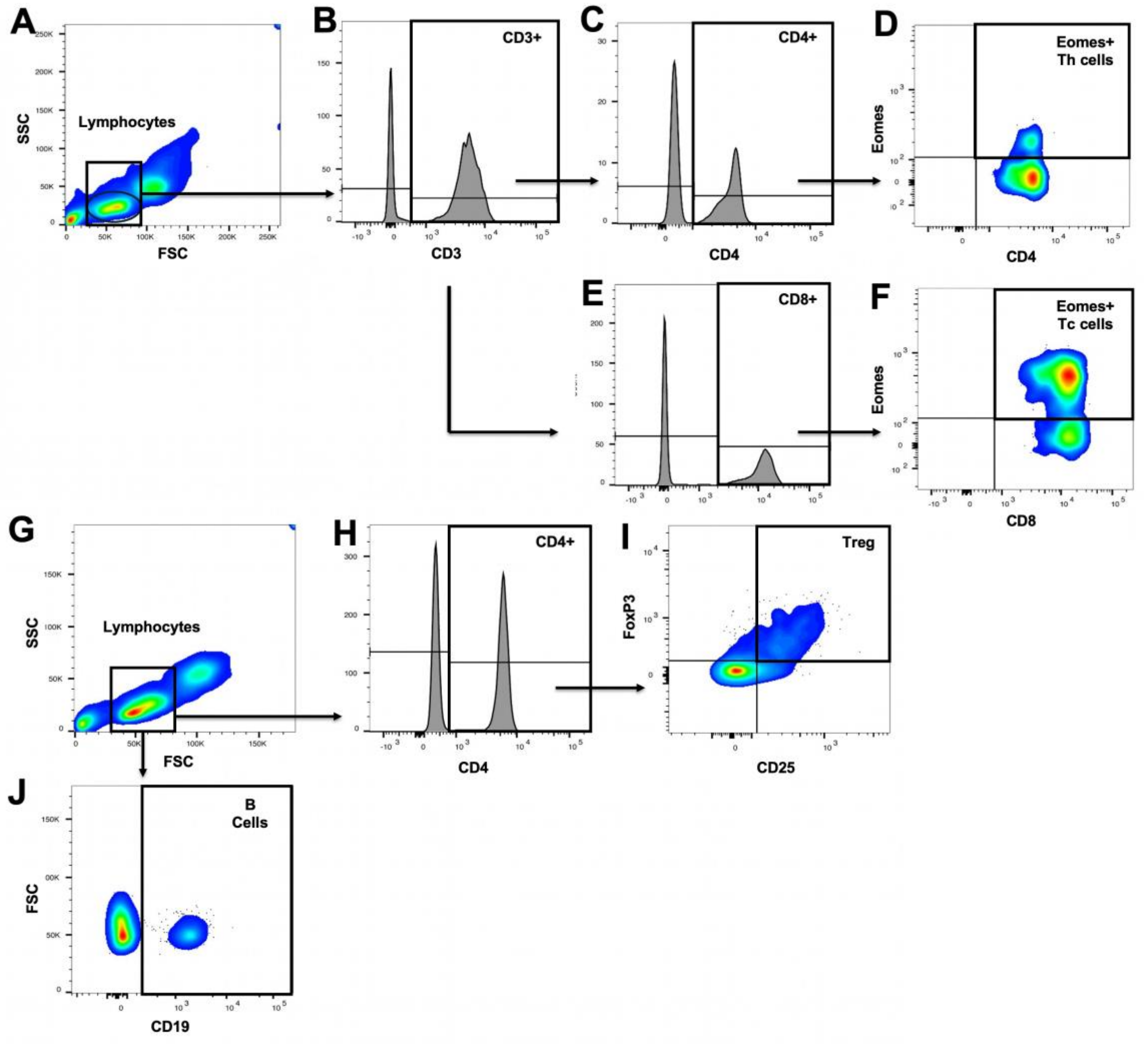

Supplementary Figure 1: Gating Strategy. a) Gating of lymphocytes via FSC/SSC. b) CD3^{+} gating for T cells. c) CD4^{+} Th cells and e) CD8^{+} Tc cells can be differentiated. d) Expression of Eomes in CD3^{+} CD4^{+} Th cells is shown. f) Expression of Eomes in CD3^{+} CD8^{+} Th cells is shown. g) Gating of lymphocytes via FSC/SSC. h) After CD4^{+} gating for Th cells, i) Treg cells are identified by simultaneous expression of CD25 and Foxp3. j) CD19^{+} gating for identifying B cells. **Abbreviations:** Eomes, Eomesodermin; FSC, forward scatter; SSC, side scatter; Tc cells, Cytotoxic T cells; Th cells, T Helper cells; Treg cells, Regulatory T cells.

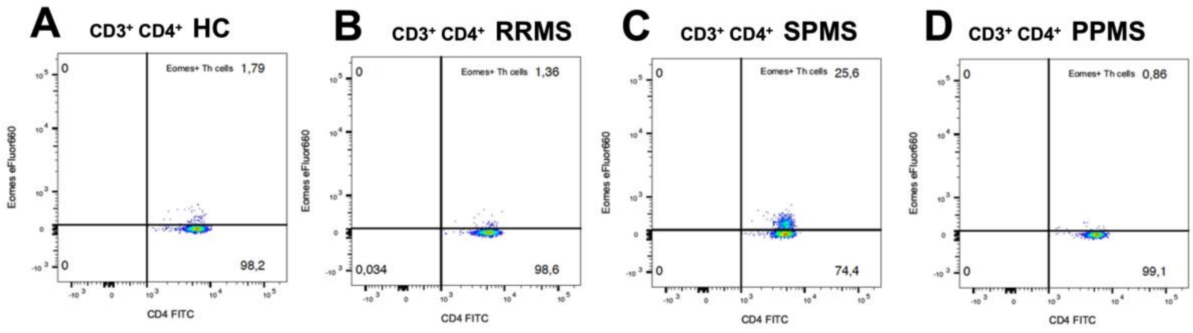

Supplementary Figure 2: Representative dot plots from flow cytometry analysis showing Eomes^{+} Th cells in healthy controls, RRMS, SPMS and PPPMS. Abbreviations: Eomes, Eomesodermin; HC, Healthy controls; PPMS, primary progressive multiple sclerosis; RRMS, relapsing remitting multiple sclerosis; SPMS, secondary progressive multiple sclerosis.

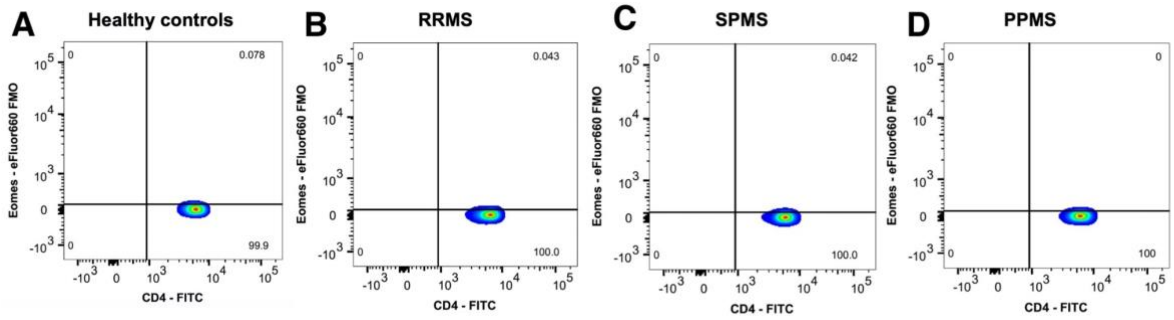

Supplementary Figure 3: FMO staining controls from healthy controls, RRMS-patients, SPMS-patients and PPMS-patients. Fluorescence-minus-one staining controls is shown for the expression of the intracellular transcription factor Eomes in a) HC, b) RRMS patients, c) SPMS patients and d) PPMS patients. Abbreviations: Eomes, Eomesodermin; PPMS, primary progressive multiple sclerosis; RRMS, relapsing remitting multiple sclerosis; SPMS, secondary progressive multiple sclerosis.

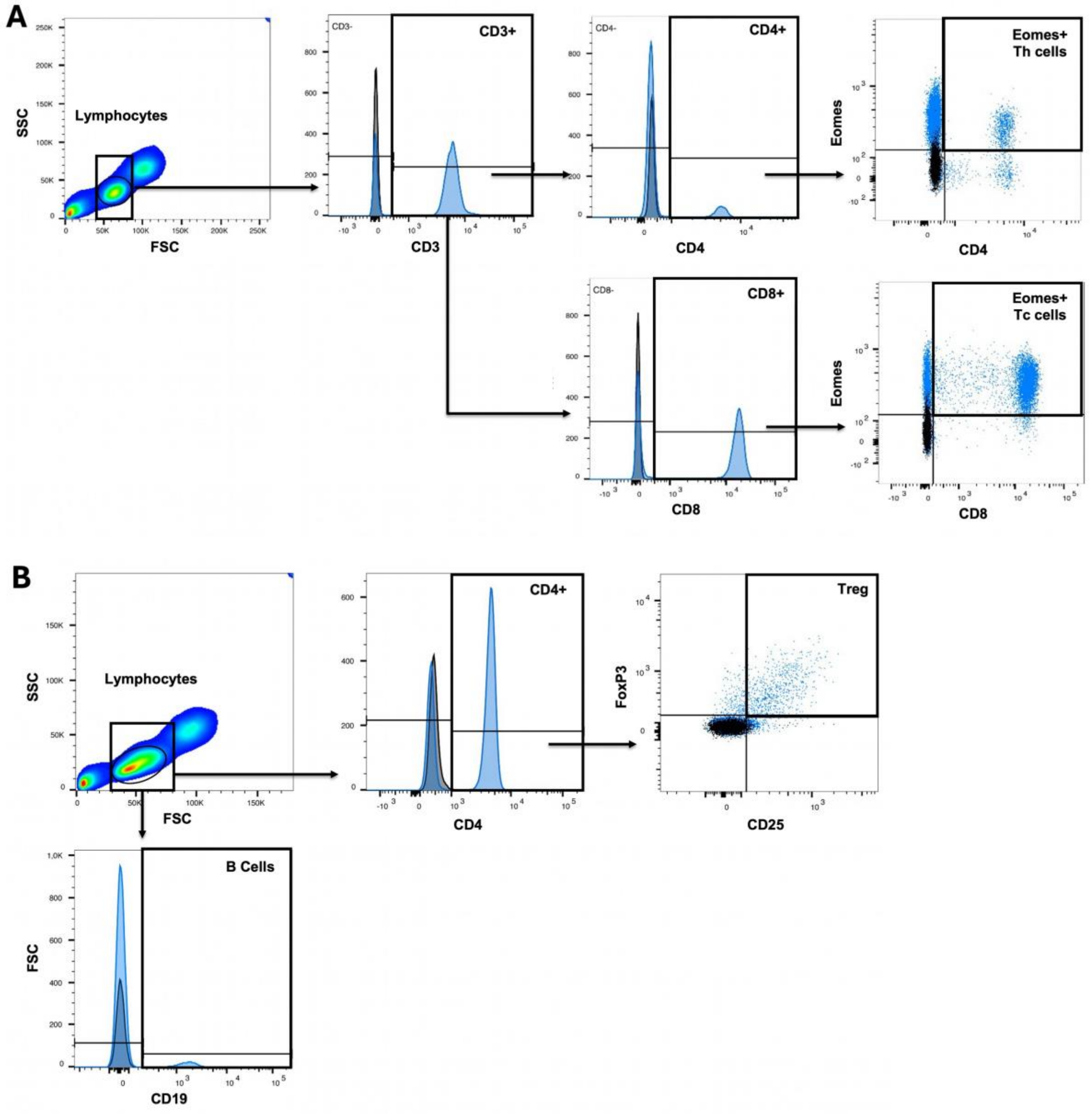

Supplementary Figure 4: Representative isotype controls for T cells and B cells. Stained sample (blue) and isotype control (black) shown for all lymphocytes. The isotype control serves as a negative reference to confirm specificity of marker expression.

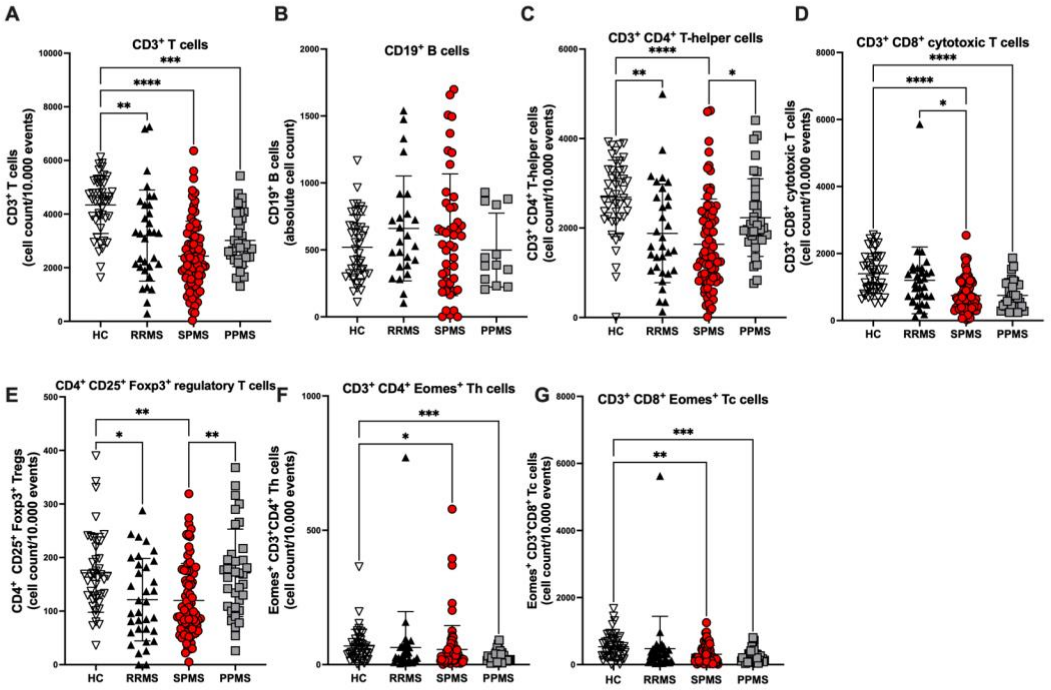

Supplementary Figure 5: Immune cell subsets in patients with multiple sclerosis (Baseline, absolute cell counts). Absolute cell counts are normalized to 10,000 acquired events for a) $CD3^+$ T cell b) B cells, c) $CD3^+$ $CD4^+$ Th cells, d) $CD3^+$ $CD8^+$ Tc cells, e) $CD4^+$ $CD25^+$ $Foxp3^+$ regulatory T cells, f) $Eomes^+$ Th cells and g) $Eomes^+$ Tc. a-g) Data are derived from n=45 HC, n=33 RRMS patients, n=79 SPMS patients and n=36 PPMS patients. b) Patients with current B cell depleting therapy are excluded from data analysis. Individual data points represent the measurement of one sample of one patient. Shown is a mean ± SD for each patient group. Data were analyzed using non-parametric Kruskal-Wallis test with post-hoc Dunn's multiple comparison test. *p<0.05, **p<0.01, ***p<0.001, ****p<0.0001. Abbreviations: Eomes, Eomesodermin; HC, healthy controls; PPMS, primary progressive multiple sclerosis; RRMS, relapsing remitting multiple sclerosis; SPMS, secondary progressive multiple sclerosis.

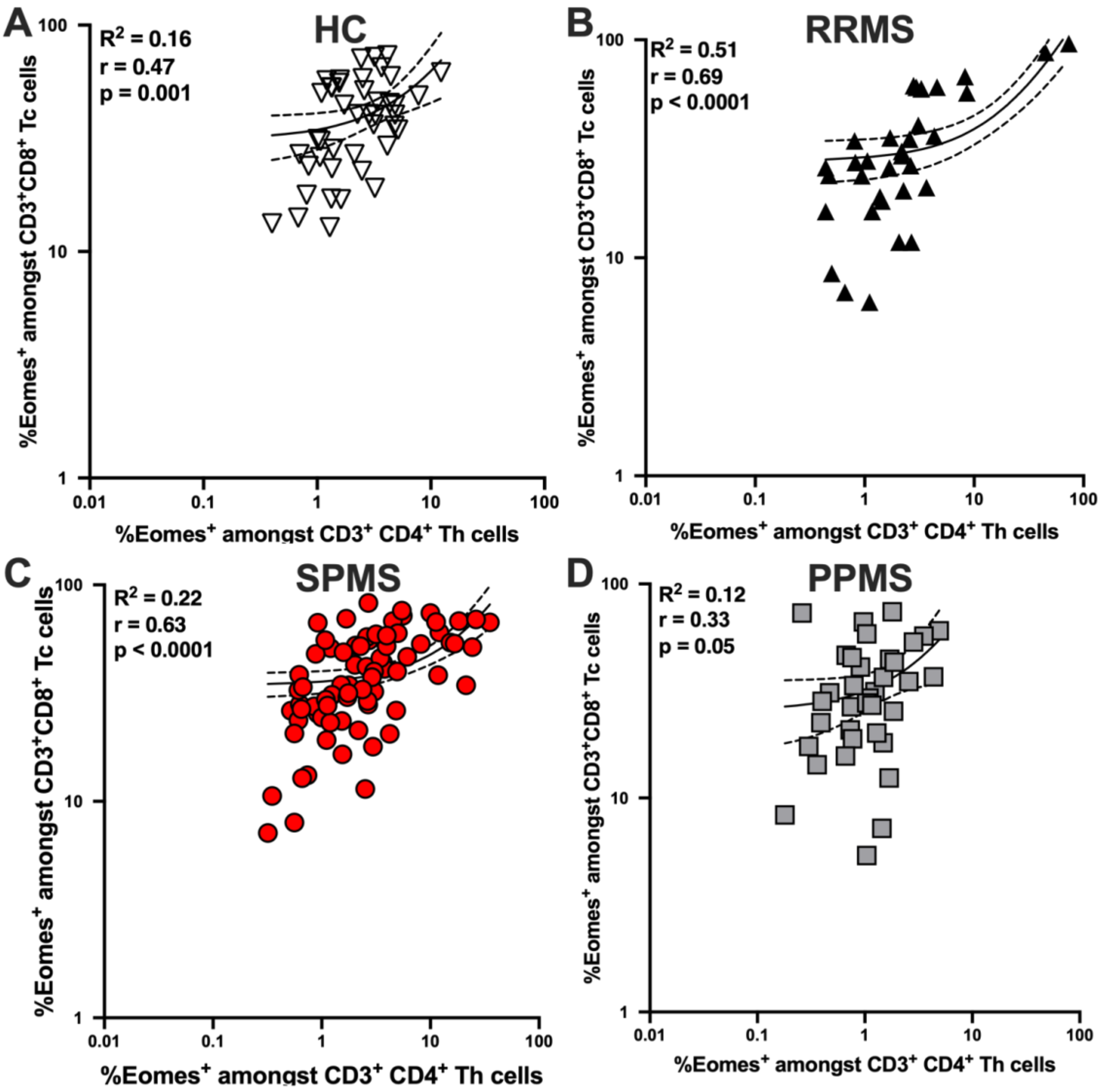

Supplementary Figure 6: Expression of Eomes is consistent in Th cells and Tc cells: Number of Eomes+ Th cells correlated with the number of Eomes+ Tc cells in a) HC (r=0.47; p=0.001), b) RRMS patients (r=0.69; p<0.0001), c) SPMS patients (r=0.63; p<0.0001) and d) PPMS patients (r=0.33; p=0.05). Data were derived from n=43 HC, n=33 RRMS patients, n=79 SPMS patients and n=36 PPMS patients. Individual data points represent the measurement of one sample of one patient. Shown is a-d) linear regression with 95% confidence interval. Data were analyzed using non-parametric Spearman test. Abbreviations: Eomes, Eomesodermin; HC, healthy controls; PPMS, primary progressive multiple sclerosis; RRMS, relapsing remitting multiple sclerosis; SPMS, secondary progressive multiple sclerosis.

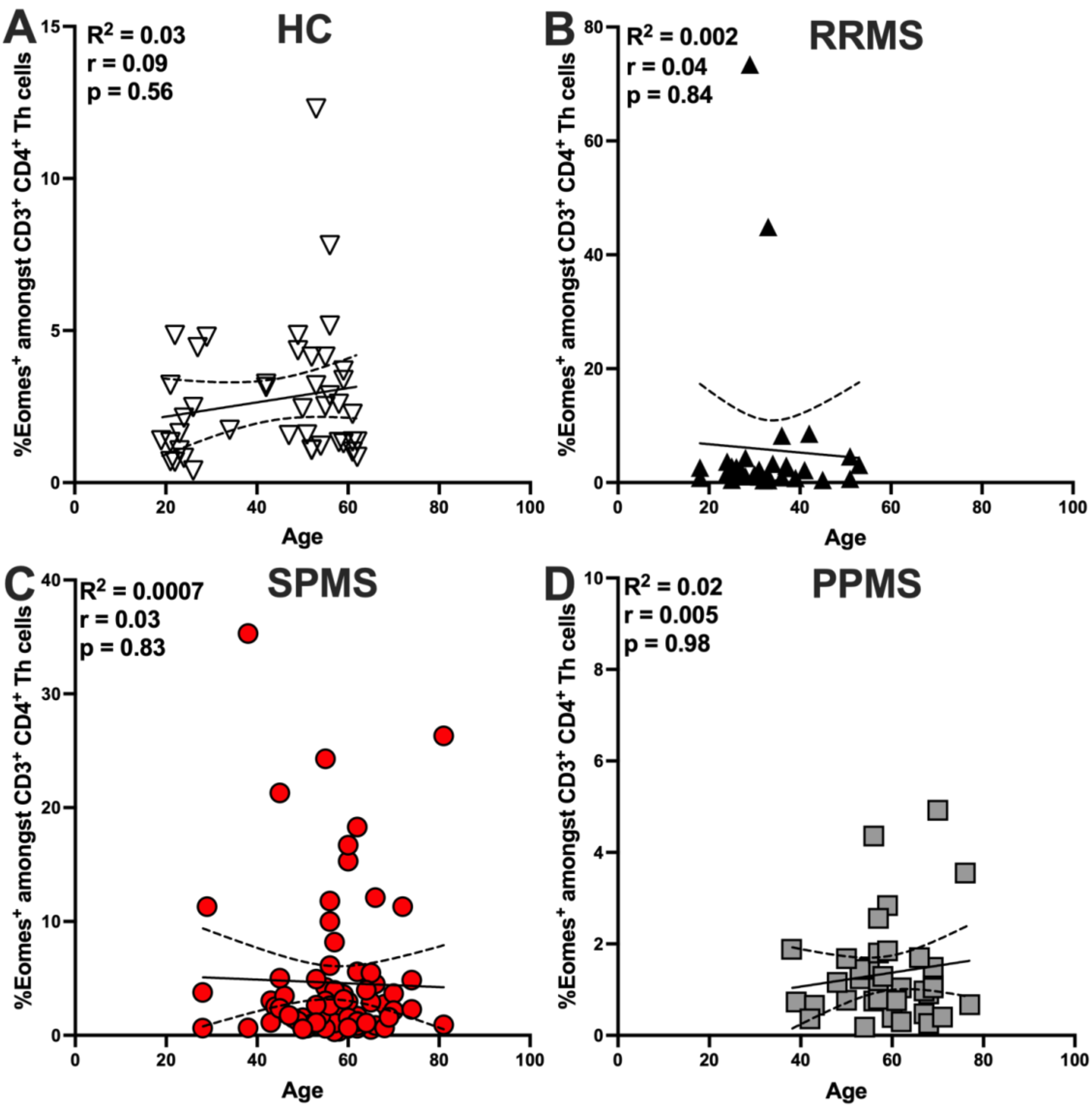

Supplementary Figure 7: Correlation of Eomes+ Th cells with age. Eomes+ Th cells did not correlate with age in a) HC, b) RRMS patients, c) SPMS patients and d) PPMS patients. Data are derived from n=45 HC, n=33 RRMS, n=79 SPMS, n=36 PPMS. Individual data points represent the measurement of one sample of one patient. Shown is a-d) linear regression with 95% confidence interval. Data were analyzed using non-parametric Spearman test. Abbreviations: Eomes, Eomesodermin; HC, healthy controls; PPMS, primary progressive multiple sclerosis; RRMS, relapsing remitting multiple sclerosis; SPMS, secondary progressive multiple sclerosis.

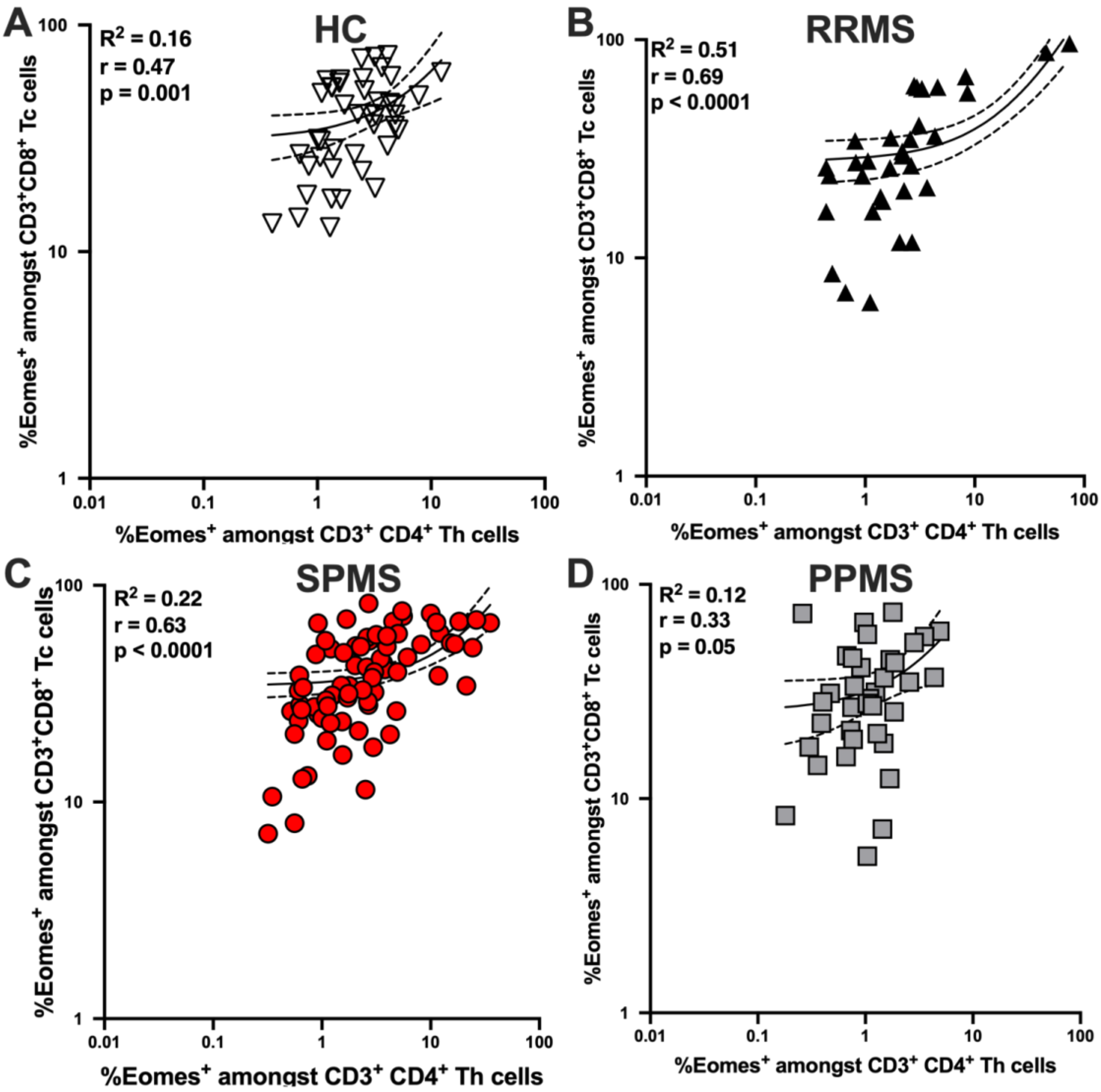

Supplementary Figure 8: Correlation of Eomes$^+$ Tc cells with age: Eomes$^+$ Tc cells did not correlate with age in a) HC and b) RRMS patients. However, Eomes$^+$ Tc cells correlated with age in c) SPMS patients (r=0.23; p=0.04) and d) PPMS patients (r=0.45; p=0.006). Data are derived from n=45 HC, n=33 RRMS, n=79 SPMS, n=36 PPMS. Individual data points represent the measurement of one sample of one patient. Shown is a-d) linear regression with 95% confidence interval. Data were analyzed using non-parametric Spearman test. Abbreviations: Eomes, Eomesodermin; HC, healthy controls; PPMS, primary progressive multiple sclerosis; RRMS, relapsing remitting multiple sclerosis; SPMS, secondary progressive multiple sclerosis.

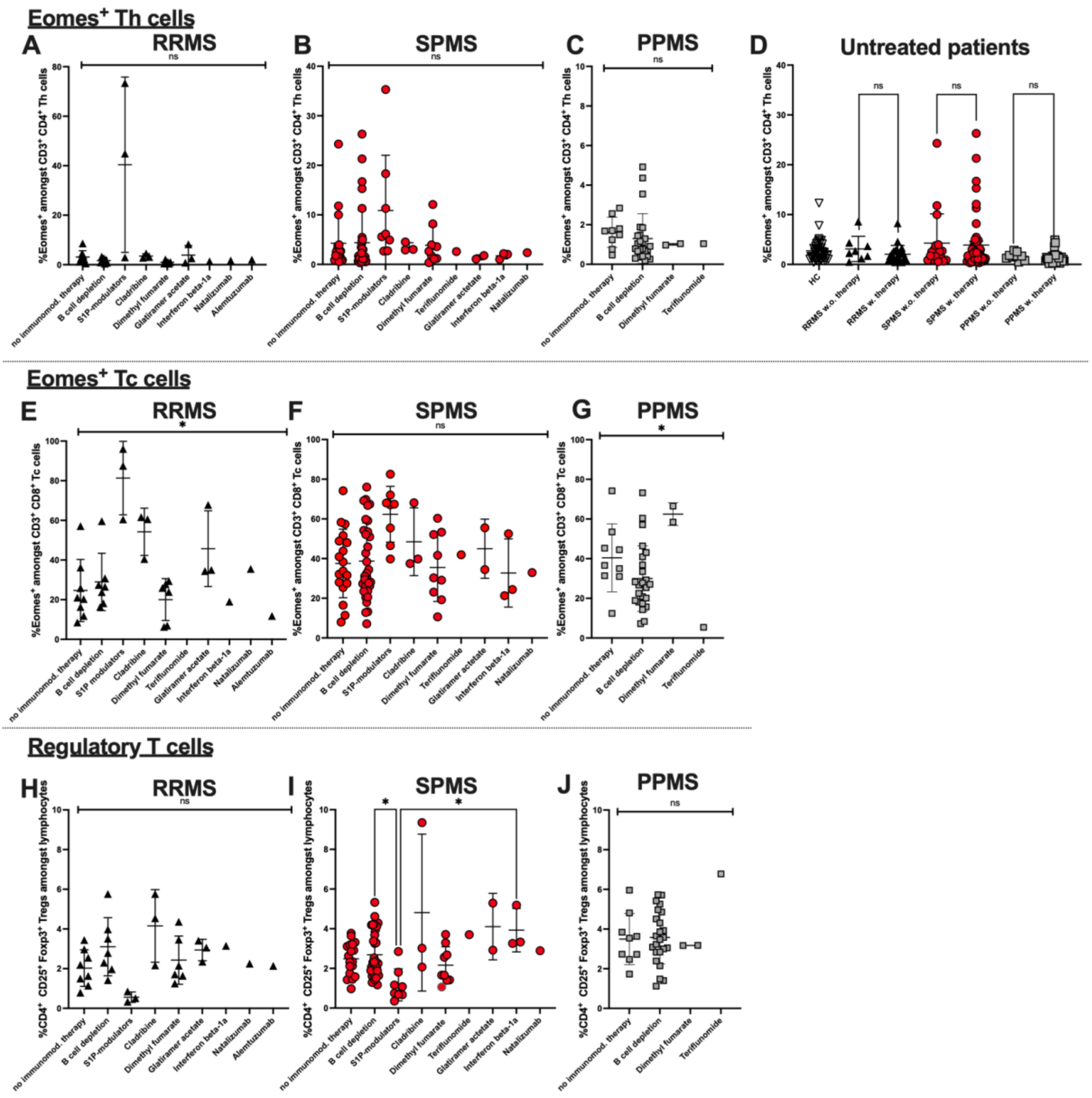

Supplementary Figure 9: Immunomodulating therapies. a – d) Eomes+ Th cells did neither differ in patients with different immunomodulating therapies nor untreated patients. e – g) Eomes+ Tc cells were not influenced by immunomodulation in f) SPMS patients. However, Eomes+ Tc cells differed in e) RRMS patients and g) PPMS patients with different therapies. h – j) Regulatory T cells were not changed in h) RRMS-patients and j) PPMS patients with immunomodulation. i) SPMS patients treated with S1P-modulators, however, showed lower frequencies of regulatory T cells compared to B cell depleted patients ($p < 0.05$) and patients treated with Interferon beta-1a ($p<0.05$). Data were derived from n=33 RRMS, n=77 SPMS, n=36 PPMS. Individual data points represent the measurement of one sample of one patient. Shown is the mean ± SD for each patient group. a-c), e-j)Data were analyzed using non-

parametric Kruskal-Wallis test with post-hoc Dunn's multiple comparison test. d) Each group was analyzed using non-parametric Mann-Whitney-Test. *p<0.05. Abbreviations: Eomes, Eomesodermin; HC, healthy controls; PPMS, primary progressive multiple sclerosis; RRMS, relapsing remitting multiple sclerosis; SPMS, secondary progressive multiple sclerosis; Tregs, Regulatory T cells.

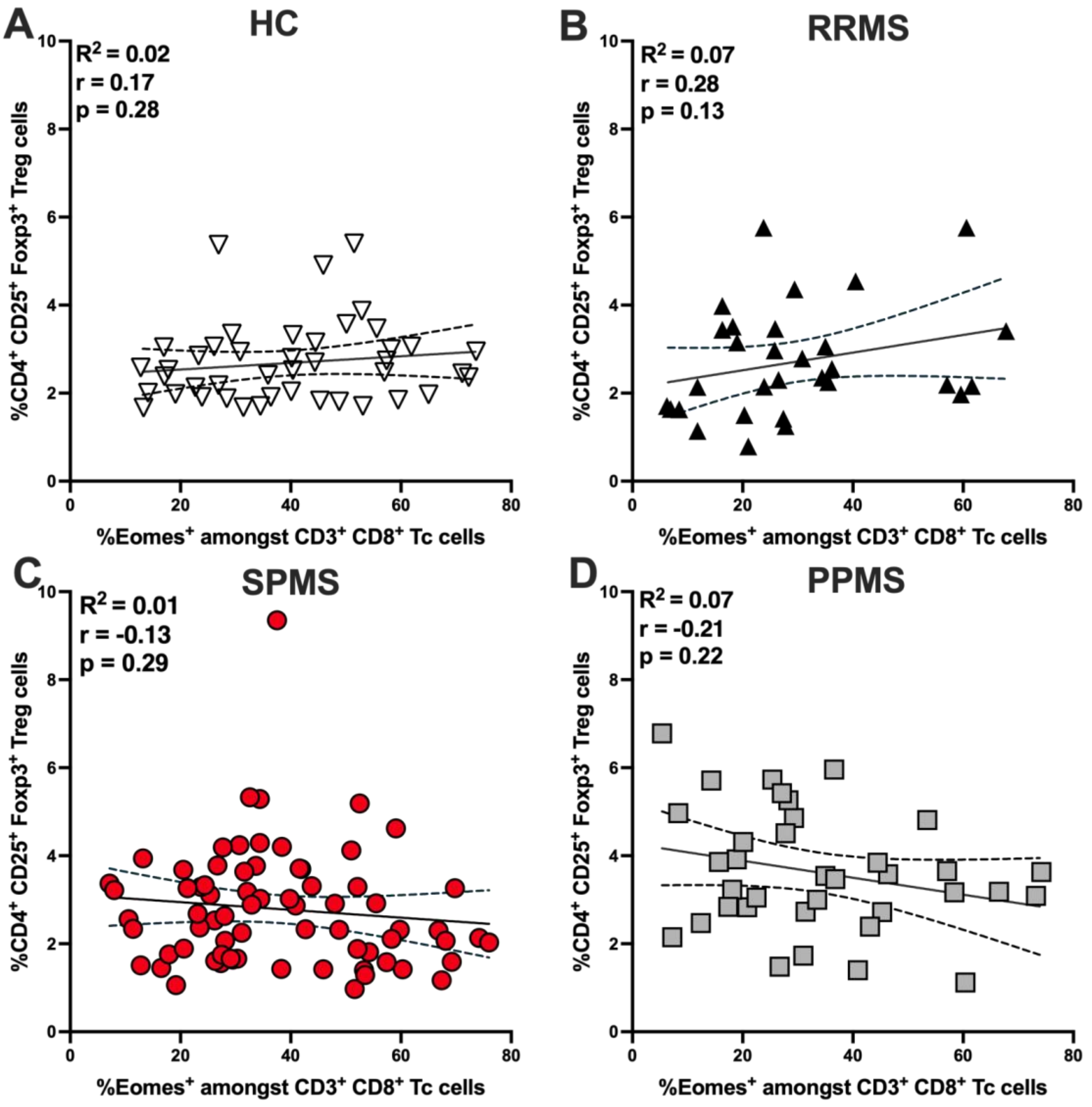

Supplementary Figure 10: No correlation between Eomes+ Tc cells and Treg cells: Number of Eomes+ Tc cells did not correlate with the number of Treg cells in a) HC, b) RRMS patients, c) SPMS patients and d) PPMS patients. RRMS patients and SPMS patients did not receive S1P-modulating therapy. Data were derived from n=43 HC, n=30 RRMS patients, n=71 SPMS patients and n=36 PPMS patients. Individual data points represent the measurement of one sample of one patient. Shown is a-d) linear regression with 95% confidence interval. Data were analyzed using non-parametric Spearman test. Abbreviations: Eomes, Eomesodermin; HC, healthy controls; PPMS, primary progressive multiple sclerosis; RRMS, relapsing remitting multiple sclerosis; SPMS, secondary progressive multiple sclerosis; Treg cells, Regulatory T cells.

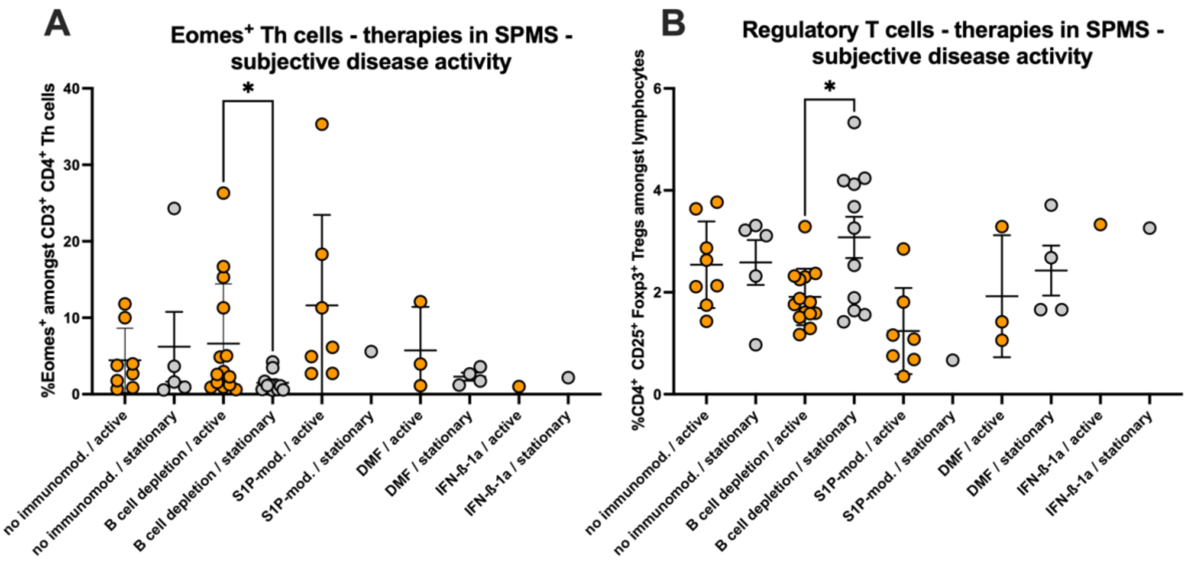

Supplementary Figure 11: Immunomodulating therapies in context with a subjective disease activity in SPMS. a) Eomes^{+} Th cells were elevated in subjectively active patients treated with B cell depletion (p=0.02) while other immunomodulating therapies did not show an effect (p>0.05). b) Regulatory T cells were equally lower in subjectively active patients treated with B cell depletion (p=0.03) while other immunomodulating therapies did not show an influence (p>0.05). Individual data points represent the measurement of one sample of one patient. Shown is a mean ± SD for each patient group. Data were analyzed using non-parametric Mann-Whitney test. Abbreviations: DMF, Dimethyl fumarate; Eomes, Eomesodermin; IFN-ß-1a, Interferon-beta-1a; S1P-mod, Sphingosine-1-phosphate receptor modulator; SPMS, secondary progressive multiple sclerosis; Treg cells, Regulatory T cells.

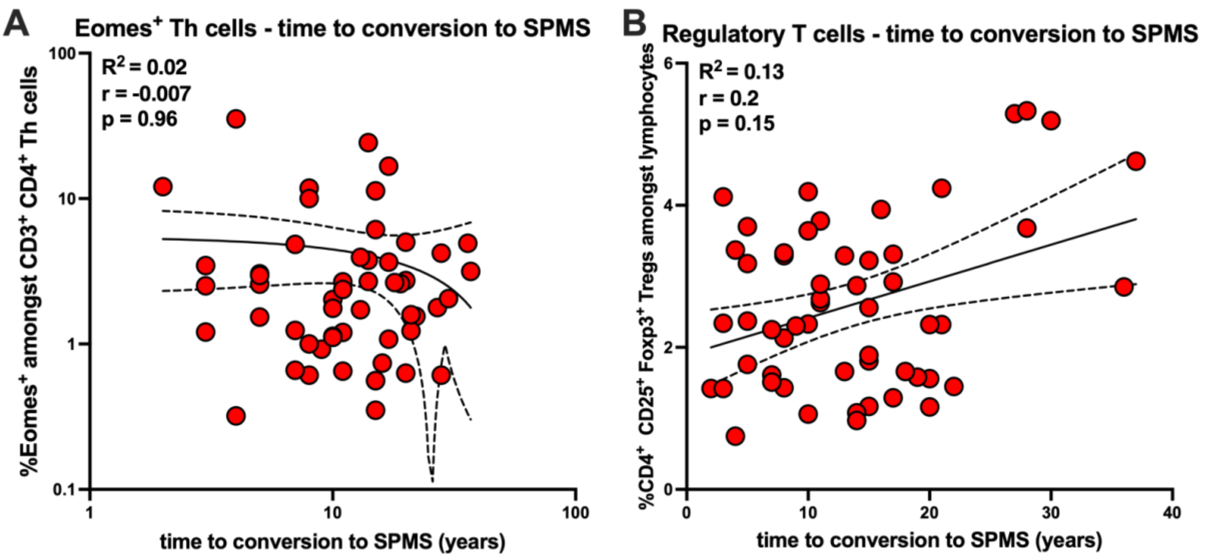

Supplementary Figure 12: Association of Eomes$^+$ Th cells and regulatory T cells with time to conversion to secondary progressive multiple sclerosis. Neither a) Eomes$^+$ Th cells nor b) regulatory T cells correlate with the time to conversion to SPMS. Data were derived from n=54 SPMS patients. Individual data points represent the measurement of one sample of one patient. Shown is a linear regression with 95% confidence interval. Data were analyzed using non-parametric Spearman test. Abbreviations: Eomes, Eomesodermin; SPMS, secondary progressive multiple sclerosis; Treg cells, Regulatory T cells.

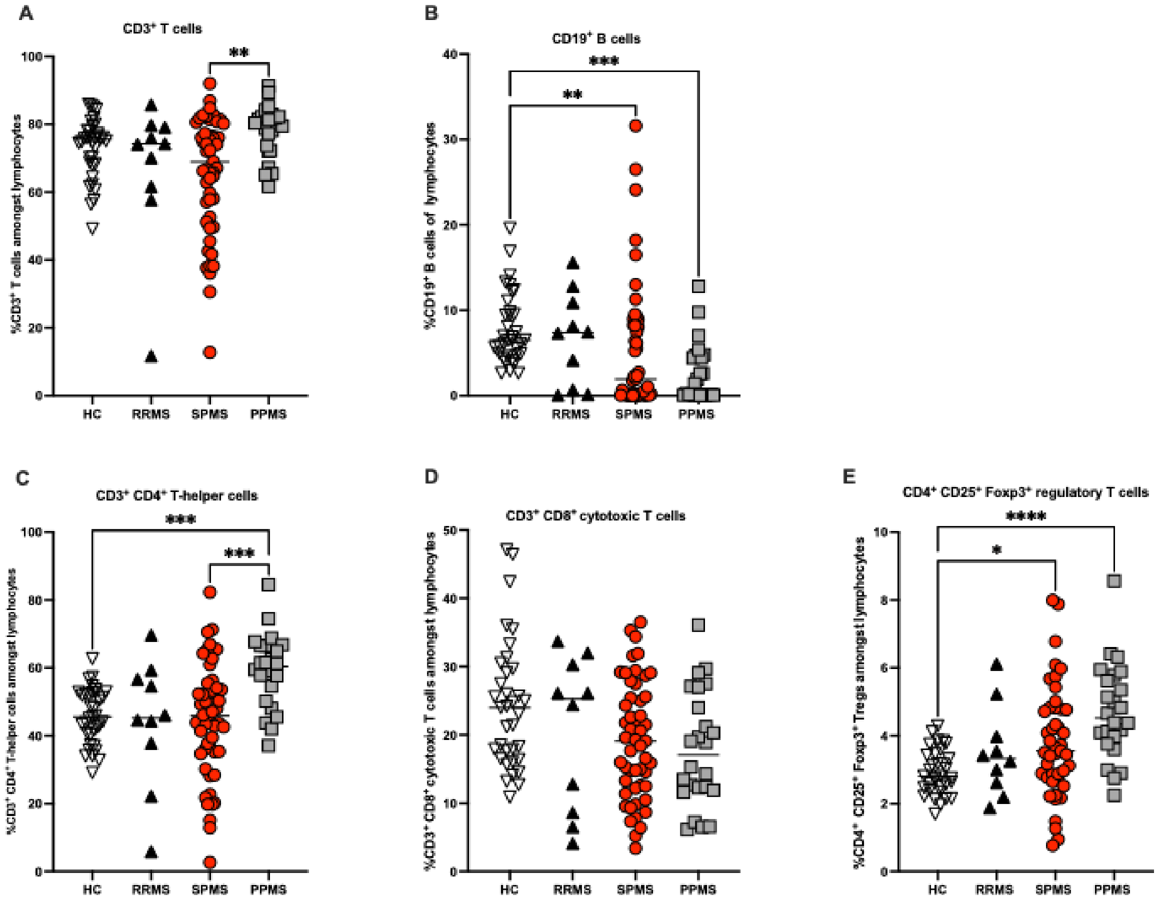

Supplementary Figure 13: Immune cell subsets in patients with multiple sclerosis (Follow up). A) CD3+ T cell were higher in PPMS patients compared to SPMS. B) B cells were lower in SPMS patients and PPMS patients compared to healthy controls. C) $CD3^+$ $CD4^+$ Th cells were elevated in PPMS patients compared to SPMS patients and healthy controls. D) Proportions of $CD3^+$ $CD8^+$ Tc cells did not differ clearly between groups. E) $CD4^+$ $CD25^+$ $Foxp3^+$ regulatory T cells were elevated in PPMS and SPMS patients compared to healthy controls. Patients receiving S1P-modulators were excluded from data analysis.Data are derived from n=35 HC, n=10 RRMS patients, n=51 SPMS patients and n=24 PPMS patients. Individual data points represent the measurement of one sample of one patient. Shown is a mean ± SD for each patient group. Data were analyzed using non-parametric Kruskal-Wallis test with post-hoc Dunn's multiple comparison test. Abbreviations: Eomes, Eomesodermin; HC, healthy controls; PPMS, primary progressive multiple sclerosis; RRMS, relapsing remitting multiple sclerosis; SPMS, secondary progressive multiple sclerosis.

References

1. C G, R D, PM T: **CAT-A Computational Anatomy Toolbox for the Analysis of Structural MRI Data.**
2. Schmidt P, Gaser C, Arsic M, Buck D, Förschler A, Berthele A, Hoshi M, Ilg R, Schmid VJ, Zimmer C, et al: **An automated tool for detection of FLAIR-hyperintense white-matter lesions in Multiple Sclerosis.** *Neuroimage* 2012, **59:**3774-3783.
3. Diedrichsen J: **A spatially unbiased atlas template of the human cerebellum.** *Neuroimage* 2006, **33:**127-138.
4. Oishi K, Faria A, Jiang H, Li X, Akhter K, Zhang J, Hsu JT, Miller MI, van Zijl PC, Albert M, et al: **Atlas-based whole brain white matter analysis using large deformation diffeomorphic metric mapping: application to normal elderly and Alzheimer's disease participants.** *Neuroimage* 2009, **46:**486-499.
